# Neuron-oligodendrocyte potassium shuttling at nodes of Ranvier protects against inflammatory demyelination

**DOI:** 10.1172/JCI164223

**Published:** 2023-04-03

**Authors:** Hannah Kapell, Luca Fazio, Julia Dyckow, Sophia Schwarz, Andrés Cruz-Herranz, Christina Mayer, Joaquin Campos, Elisa D’Este, Wiebke Möbius, Christian Cordano, Anne-Katrin Pröbstel, Marjan Gharagozloo, Amel Zulji, Venu Narayanan Naik, Anna Delank, Manuela Cerina, Thomas Müntefering, Celia Lerma-Martin, Jana K. Sonner, Jung Hyung Sin, Paul Disse, Nicole Rychlik, Khalida Sabeur, Manideep Chavali, Rajneesh Srivastava, Matthias Heidenreich, Kathryn C. Fitzgerald, Guiscard Seebohm, Christine Stadelmann, Bernhard Hemmer, Michael Platten, Thomas J. Jentsch, Maren Engelhardt, Thomas Budde, Klaus-Armin Nave, Peter A. Calabresi, Manuel A. Friese, Ari J. Green, Claudio Acuna, David H. Rowitch, Sven G. Meuth, Lucas Schirmer

**Affiliations:** 1 Department of Neurology, Medical Faculty Mannheim, Heidelberg University, Heidelberg, Germany.; 2 Department of Neurology with Institute of Translational Neurology, University Hospital Münster (UKM), Münster, Germany.; 3 Department of Neurology, University of Düsseldorf, Dusseldorf, Germany.; 4 Weill Institute for Neurosciences, Department of Neurology, UCSF, San Francisco, California, USA.; 5 Institute of Neuroimmunology and Multiple Sclerosis, Center for Molecular Neurobiology Hamburg, University Medical Center Hamburg-Eppendorf (UKE), Hamburg, Germany.; 6 Chica and Heinz Schaller Research Group, Institute of Anatomy and Cell Biology, Heidelberg University, Heidelberg, Germany.; 7 Optical Microscopy Facility, Max Planck Institute for Medical Research, Heidelberg, Germany.; 8 Electron Microscopy Core Unit, Department of Neurogenetics, Max Planck Institute of Experimental Medicine, Göttingen, Germany.; 9 Cluster of Excellence, “Multiscale Bioimaging: from Molecular Machines to Network of Excitable Cells” (MBExC), University of Göttingen, Göttingen, Germany.; 10 Neurologic Clinic and Policlinic and Research Center for Clinical Neuroimmunology and Neuroscience Basel, Departments of Medicine, Biomedicine, and Clinical Research, University Hospital of Basel, University of Basel, Basel, Switzerland.; 11 Departments of Neurology and Neuroscience, Johns Hopkins University School of Medicine, Baltimore, Maryland, USA.; 12 Institute for Genetics of Heart Diseases (IfGH), Cellular Electrophysiology and Molecular Biology, UKM, Münster, Germany.; 13 University of Münster, Chembion, Münster, Germany.; 14 Institute of Physiology I, University of Münster, Münster, Germany.; 15 Eli and Edythe Broad Center of Regeneration Medicine and Stem Cell Research and; 16 Department of Pediatrics, UCSF, San Francisco, California, USA.; 17 Department of Neurology, School of Medicine, Technical University of Munich, Munich, Germany.; 18 Leibniz-Forschungsinstitut für Molekulare Pharmakologie (FMP) and Max-Delbrück-Centrum für Molekulare Medizin (MDC), Berlin, Germany.; 19 Institute of Neuropathology, University Medical Center, Göttingen, Germany.; 20 Munich Cluster for Systems Neurology, Munich, Germany.; 21 DKTK Clinical Cooperation Unit Neuroimmunology and Brain Tumor Immunology, German Cancer Research Center (DKFZ), INF 280, Heidelberg, Germany.; 22 Interdisciplinary Center for Neurosciences (IZN) and; 23 Mannheim Center for Translational Neuroscience and Institute for Innate Immunoscience, Medical Faculty Mannheim, Heidelberg University, Heidelberg, Germany.; 24 Neurocure Cluster of Excellence, Charité University Medicine Berlin, Berlin, Germany.; 25 Institute of Neuroanatomy, Medical Faculty Mannheim, Heidelberg University, Heidelberg, Germany.; 26 Institute of Anatomy and Cell Biology, Johannes Kepler University Linz, Linz, Austria.; 27 Department of Ophthalmology, UCSF, San Francisco, California, USA.; 28 Wellcome Trust–Medical Research Council Stem Cell Institute and; 29 Department of Paediatrics, University of Cambridge, Cambridge, United Kingdom.; 30 Department of Neurosurgery, UCSF, San Francisco, California, USA.

**Keywords:** Inflammation, Neuroscience, Multiple sclerosis, Neurodegeneration, Potassium channels

## Abstract

Multiple sclerosis (MS) is a progressive inflammatory demyelinating disease of the CNS. Increasing evidence suggests that vulnerable neurons in MS exhibit fatal metabolic exhaustion over time, a phenomenon hypothesized to be caused by chronic hyperexcitability. Axonal Kv7 (outward-rectifying) and oligodendroglial Kir4.1 (inward-rectifying) potassium channels have important roles in regulating neuronal excitability at and around the nodes of Ranvier. Here, we studied the spatial and functional relationship between neuronal Kv7 and oligodendroglial Kir4.1 channels and assessed the transcriptional and functional signatures of cortical and retinal projection neurons under physiological and inflammatory demyelinating conditions. We found that both channels became dysregulated in MS and experimental autoimmune encephalomyelitis (EAE), with Kir4.1 channels being chronically downregulated and Kv7 channel subunits being transiently upregulated during inflammatory demyelination. Further, we observed that pharmacological Kv7 channel opening with retigabine reduced neuronal hyperexcitability in human and EAE neurons, improved clinical EAE signs, and rescued neuronal pathology in oligodendrocyte–Kir4.1–deficient (OL-Kir4.1–deficient) mice. In summary, our findings indicate that neuron-OL compensatory interactions promoted resilience through Kv7 and Kir4.1 channels and identify pharmacological activation of nodal Kv7 channels as a neuroprotective strategy against inflammatory demyelination.

## Graphical abstract

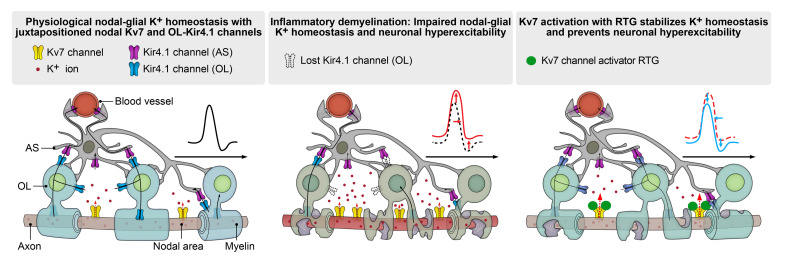

## Introduction

Multiple sclerosis (MS) is the most prevalent inflammatory disease of the CNS with an accumulating lesion load over time, ongoing brain atrophy, and progressive neuronal injury (1). Axonal damage and, eventually, loss of neurons are hallmarks of pathology in progressive MS (2–4), however, little is known about the molecular mechanisms underlying the chronic neuronal dysfunction caused by changes in excitability (5). For example, sustained damage to the node of Ranvier (NoR) during inflammatory demyelination results in altered ion channel distribution with direct effects on axonal conduction and neuronal excitability (6). Although changes in neuronal excitability have been described in MS animal models, such as that of experimental autoimmune encephalomyelitis (EAE) (7) and other in vivo models of de- and remyelination (8–11), rational treatment strategies to normalize the levels of neuronal excitability to prevent neurodegeneration are lacking.

We previously found that excitatory cortical projection neurons exhibit a high level of “transcriptional” dysregulation in MS suggestive of metabolic exhaustion and ion imbalance (12). In particular, sodium channels exhibit a diffusely altered expression pattern along demyelinated axons (13), which contributes to changes in neuronal excitability (14, 15) and suggest ion imbalance as an interesting therapeutic target in MS (16, 17). Other studies reported that mitochondrial impairment and energy deficiency result in ion gradient breakdown and calcium overload, which further increases the risk for neuronal injury (18, 19). Hence, one intriguing, but so far unproven, hypothesis is that chronic hyperexcitability is a key driver of neuronal vulnerability in MS (20). The enhanced neuronal excitability is likely the consequence of various factors that lower the threshold to generate action potentials in the context of chronic inflammatory demyelination.

For example, because of its role in potassium/K^+^ buffering at periaxonal and paranodal spaces, oligodendroglial Kir4.1 channels, expressed at inner and outer myelin tongues and paranodes (21), are critical for stabilizing neuronal excitability and maintaining function. Independently, loss of either astroglial or oligodendroglial Kir4.1 channel function has been shown to increase neuronal excitability, thus triggering seizures and destabilizing axonal integrity (21–24). However, it remains unclear how oligodendroglial ion channel dysfunction mechanistically drives neuronal pathology during disease progression.

As opposed to para- and juxtanodal Kir4.1 channels, neuronal Kv7 channels are mainly responsible for outward K^+^ currents and membrane repolarization at the NoR and the axon initial segment (AIS) (25, 26), regulating the threshold and frequency of action potential discharge (27). In most neurons, M-channels consist only of Kv7.2 and Kv7.3 subunits, making them the most important Kv7 subunits to regulate neuronal excitability (26, 28), which can be activated by the small molecule retigabine (RTG), (29, 30). RTG is a specific Kv7 channel opener that has shown beneficial effects in hyperexcitability-related disorders such as experimental seizure and chronic pain models (31–34), and has been studied in the treatment of human epilepsy and motor neuron diseases (32, 35–37). These beneficial effects of RTG are attributed to stabilization of the neuronal resting membrane potential, which results in reduced neuronal excitability. On the basis of clinical trials (38–40), which demonstrated efficacy and tolerability despite side effects like skin and retina pigmentation with previous formulations, RTG was initially approved in 2011 by the FDA and the European Medicines Agency (EMA) for adjunctive treatment of partial-onset seizures in adult patients.

Here, we describe a functional interplay of nodal Kv7 and neighboring OL-Kir4.1 K^+^ channels in the context of K^+^ shuttling, both of which we found to be dysregulated during inflammatory demyelination. Specifically, OL-Kir4.1 channels were downregulated and Kv7 channel subunits were transiently upregulated during the course of human MS and mouse EAE, suggesting a compensatory mechanism in neurons to counteract hyperexcitability and increase resilience. We also found that early treatment with RTG assuaged neuronal degeneration and improved clinical outcomes in EAE and an OL-Kir4.1 loss-of-function model. Our findings suggest that neuron-OL K^+^ shuttling at the NoR represents a druggable interface to protect neurons against inflammatory demyelination.

## Results

### Ultrastructural mapping of Kv7 and Kir4.1 channels at NoR.

**Figure 1:**
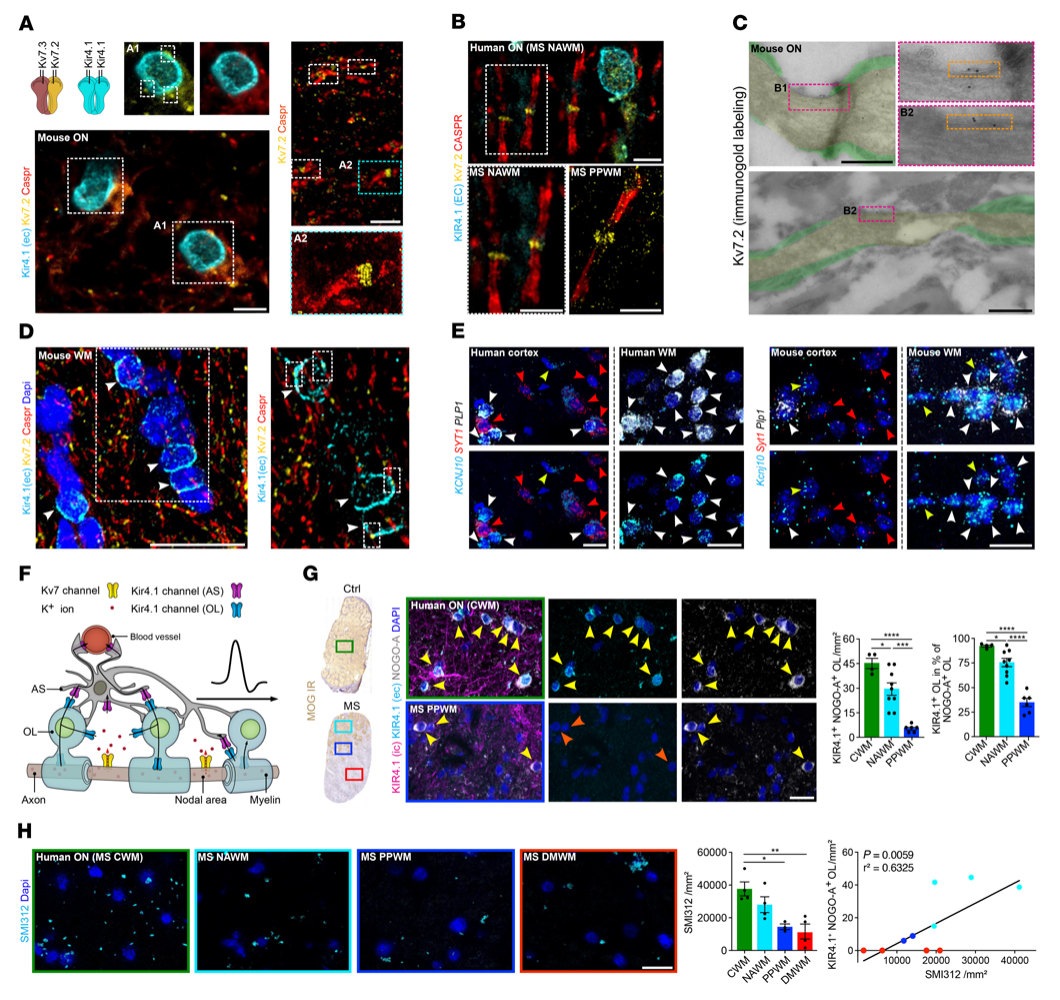
Investigation of Kir4.1 and Kv7 channels in neuroglial cell types under homeostatic and inflammatory demyelinating conditions. (**A** and **B**) Triple staining (Caspr, Kir4.1 [extracellular epitope, EC], Kv7.2) revealed specific nodal expression of Kv7.2 (flanked by Caspr IR) adjacent to OL-Kir4.1 channel IR in mouse (**A**) and human (**B**) ON. A2 close-up (stimulated emission depletion [STED] image) shows the approximately 190 nm periodic organization of Kv7.2. (**C**) Kv7.2 immunogold EM labeling shows the presence of gold particles in nodal areas (yellow) between myelin sheets (green) in control mouse ON. (**D**) Triple staining (compare with **A**) confirmed the juxtapositioning of OL-Kir4.1 and nodal Kv7.2 channels (white arrows) in other mouse WM tracts (corpus callosum). (**E**) Perineuronal mouse *Kcnj10* and human *KCNJ10* expression (ISH) was visualized in mouse and human cortex with OLs coexpressing *Plp1*^+^ (mouse) or *PLP1*^+^ (human) and *Kcnj10*^+^ or *KCNJ10*^+^ (white arrowheads) next to *Syt1*^+^, *SYT1*^+^, *Kcnj10*^–^, and *KCNJ10*^–^ neurons (red arrowheads). Yellow arrowheads indicate *Kcnj10*^+^, *KCNJ10*^+^, *Plp1*^–^, and *PLP1*^–^ astrocytes. (**F**) Cartoon illustrates neuron-OL for the K^+^ shuttling mechanism: neuronal Kv7 channels mediate axonal K^+^ efflux, and OL-Kir4.1 channels mediate extracellular K^+^ uptake and siphoning through interaction with astrocyte (AS) Kir4.1 channels. (**G**) In human MS ON, KIR4.1 channel IR (antibodies against intracellular [specific for OL-KIR4.1 and AS-KIR4.1] and extracellular [specific for OL-KIR4.1] epitopes) was preserved on astrocyte fibers in lesions. OL-KIR4.1 channel IR (yellow arrows) was reduced in MS NAWM areas (*n* = 9) and lost in PPWM (*n* = 6) relative to CWM (*n* = 4) based on MOG IR. (**H**) SMI312^+^ axon density was gradually lost in MS ON tissues toward the lesion rim and correlated with OL-KIR4.1 channel loss. Scale bars: 5 μm (**A** and **B**); 0.5 μm (**C**); 20 μm (**D** and **E**, and **G**); 100 μm (**H**). Original magnification, ×100 (enlarged insets in **A** and **B**) and ×63 (enlarged insets in **D**). *P < 0.05, **P < 0.01, ***P < 0.001, and ****P < 0.0001, by 1-way ANOVA (**G** and **H**, left) and simple linear regression (**H**, right).

We investigated the localization of neuronal Kv7 and OL-Kir4.1 channels in human and mouse optic nerve (ON) and subcortical white matter (WM) tissues (Figure 1, A–E). Kv7.2 subunits localized to nodal areas flanked by contactin-associated protein 1 (Caspr1) and in close to proximity to juxtanodal OL-Kir4.1 channels (Figure 1, A, B, and D). By electron microscopy (EM), we could confirm Kv7.2 mapping to the NoR in the mouse ON (Figure 1C). Notably, we observed a periodic arrangement of Kv7 (~190 nm; Figure 1A), in line with previous work (41). Furthermore, analysis of human control and MS tissues confirmed juxtapositioning of OL-KIR4.1 and NoR-Kv7.2 channels in normal-appearing WM (NAWM) and periplaque WM (PPWM) lesion rim areas of ON tissues from patients with MS based on myelin oligodendrocyte glycoprotein (MOG) immunoreactivity (IR) (Figure 1B and Supplemental Table 1; supplemental material available online with this article; https://doi.org/10.1172/JCI164223DS1). Using RNA ISH, we confirmed high expression levels of potassium inwardly-rectifying channel, subfamily J, member 10 (*KCNJ10*), which encodes KIR4.1, in both mouse and human subcortical WM OLs coexpressing proteolipid protein 1 (*PLP*) (Figure 1E). Of note, in cortical gray matter (GM) areas, we observed a spatial relationship between synaptotagmin-expressing (*SYT1*-expressing) neurons and *KCNJ10*-expressing OLs (Figure 1E).

In summary, using different imaging parameters, we could decipher the spatial relationship between OL-KIR4.1 and neuronal Kv7 channels across species and CNS areas, suggesting a functional crosstalk between both channels in the context of neuron-OL K^+^ shuttling and homeostasis (Figure 1F).

### OL-KIR4.1 downregulation at MS lesion rims and worsened EAE in animals with ablated OL-Kcnj10 function.

To study KIR4.1 channel IR in MS ON samples, we focused on lesion rim areas and observed a reduction of OL-KIR4.1 IR at NAWM and PPWM areas (average MS disease duration of approximately 25 years) relative to controls (Figure 1G). Similarly, we detected a loss of OL-Kir4.1 IR in chronic EAE ON tissues (45 days post induction [dpi]). Next, we assessed axonal pathology in MS ON tissue and found a gradual loss of SMI312^+^ axons toward the lesion center in a pattern similar to that observed for OL-KIR4.1 IR (Figure 1H). To investigate the consequences of ablated OL-*Kcnj10* function, we examined *Cnp-Cre Kir4.1^fl/fl^* animals in the context of EAE. We noted clinical worsening and a high frequency of epileptic seizures as EAE progressed, together with higher mortality rates in OL-*Kcnj10* conditional-knockout (cKO) mice relative to controls (Supplemental Figure 1A). OL-*Kcnj10*–deficient mice showed increased numbers of Iba1^+^ myeloid cells in spinal cord (SC) but not ON tissue samples, where we found an increase in SMI312^+^ axon caliber size (Supplemental Figure 1, B and C).

### Kv7 channel dysregulation in human MS and mouse EAE tissues.

**Figure 2:**
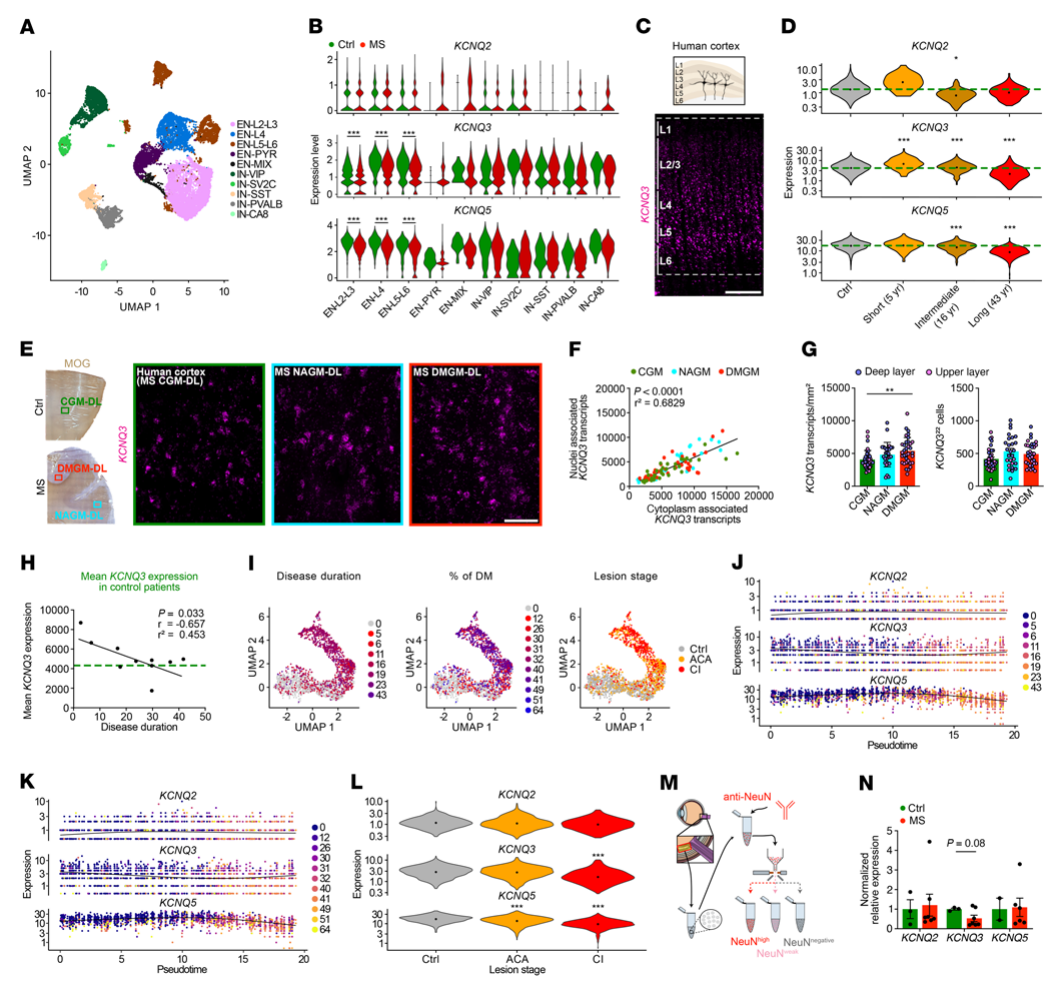
*KCNQ*3 dysregulation in cortical and retinal MS tissues. (**A**) Uniform manifold approximation and projection (UMAP) plot visualizes clustering of human excitatory (EN) and inhibitory (IN) cortical neurons based on a published snRNA-Seq data set (12). (**B**) Normalized *KCNQ2/-3/-5* expression in control (Ctrl) and MS human cortical neurons. (**C**) Spatial *KCNQ3* expression (ISH) in the human cortex. (**D**) Violin plots visualize average *KCNQ2/-3/-5* expression levels (snRNA-Seq, **A**) in control neurons (*n* = 5; green dashed line) and representative MS samples from patients with various disease durations. (**E**) *KCNQ3* ISH in human CGM and MS NAGM and DMGM lesion areas based on MOG IR. DL, deep layers. (**F**) Correlation of nucleus- and cytoplasm-associated *KCNQ3* transcript counts within the same cell in human cortical tissues (ISH) from CGM (*n* = 35 areas, *n* = 5 patients), NAGM (*n* = 27 areas, *n* = 8 patients) and DMGM (*n* = 34 areas, *n* = 8 patients). (**G**) *KCNQ3* upregulation in DMGM (ISH) independent of neuronal density. (**H**) Gradual loss of mean *KCNQ3* expression in MS GM tissues (ISH) from patients with a prolonged MS disease duration approached CGM expression levels (*n* = 5, green dashed line). (**I**) Unsupervised trajectory inference of upper L2/-3 neuron branch and nuclei distribution along the trajectory (compare with Supplemental Figure 3, E and F) based on MS disease duration, demyelination (DM) extent, and lesion stage. (**J** and **K**) Pseudotime-dependent *KCNQ2/-3/-5* expression in relation to disease duration and demyelination based on MOG IR. (**L**) Neuronal *KCNQ2/-3/-5* expression grouped by lesion stage. (**M**) Sorting of retinal nuclei based on NeuN IR. (**N**) Normalized *KCNQ2/-3/-5* expression by qPCR in human RGC nuclei (controls, *n* = 6; MS, *n* = 7). Scale bars: 500 μm (**C**); 100 μm (**E**). *P < 0.05, **P < 0.01, ***P < 0.001, by Wilcoxon rank-sum test with Bonferroni’s correction (**B**); generalized linear model by Wald test with Benjamini and Hochberg correction (**D** and **L**); simple linear regression (**F** and **H**); Kruskal-Wallis test (**G**); and mixed-effects model with Geisser-Greenhouse correction and Šidák’s multiple-comparison test (**N**). ACA, acute chronic active; CI, chronic inactive.

**Figure 3:**
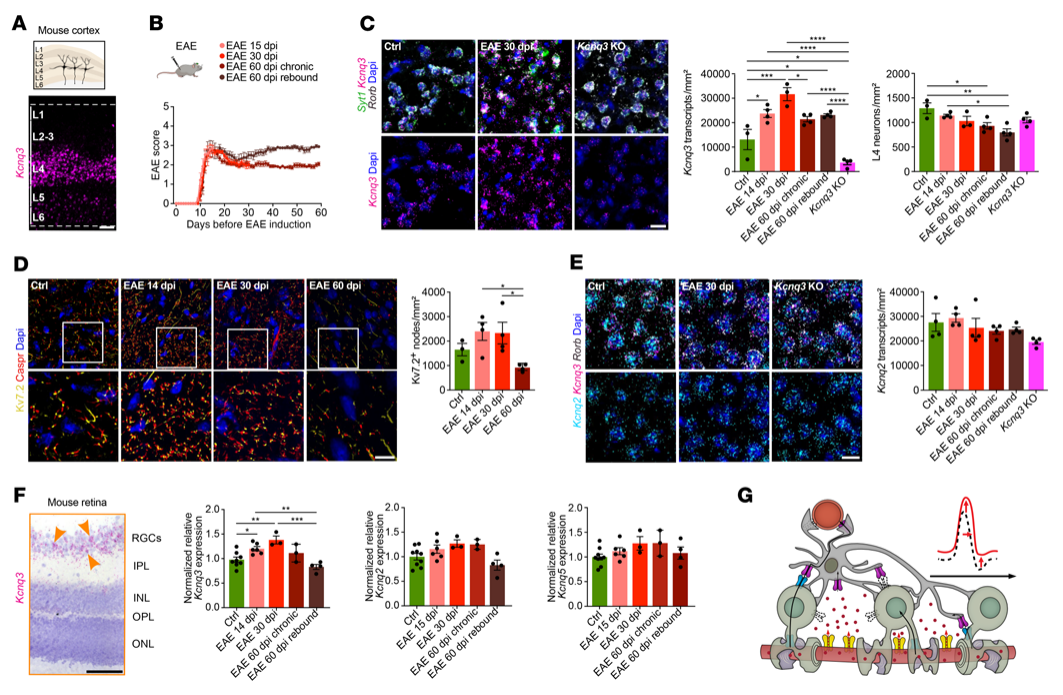
Dysregulation of Kv7 subunits in cortical and retinal EAE tissues. (**A**) Spatial *Kcnq3* expression (ISH) in the mouse cortex. (**B**) Overview plot visualizes different EAE groups including endpoints at 15, 30, and 60 dpi; note chronic EAE groups (endpoint at 60 dpi) were divided into 2 groups separating animals with or without clinical worsening (rebound). (**C**) *Kcnq3* expression (ISH) in *Syt1^+^ Rorb^+^* L4 mouse neurons at 14, 30, and 60 dpi in EAE (each, *n* = 4) and control (*n* = 3) mice; *Kcnq3*-KO mouse tissue showed a strong reduction in *Kcnq3* expression (*n* = 4). (**D**) Density of Kv7.2^+^ nodes (framed by Caspr^+^ IR) based on IR in L4 cortical areas at 14, 30, and 60 dpi in EAE (each, *n* = 4) and control (*n* = 3) tissues. (**E**) *Kcnq2* expression based on ISH in L4 mouse neurons at 14, 30, and 60 dpi in EAE, control, and *Kcnq3*-KO (each, *n* = 4) mice. (**F**) *Kcnq3* expression (ISH) of mouse retinal specimens comprising the inner plexiform layer (IPL), inner nuclear layer (INL), outer plexiform layer (OPL), and outer nuclear layer (ONL) revealed specific *Kcnq3* expression in RGCs (yellow arrows). Plots show normalized *Kcnq2/-3/-5* expression (qPCR) in sorted mouse NeuN^hi^ RGC nuclei (control, *n* = 9; 15 dpi, *n* = 6; 30 dpi, *n* = 3; 60 dpi chronic, *n* = 3; 60 dpi rebound, *n* = 4). (**G**) Cartoon illustrates dysregulated neuron-OL K^+^ shuttling during inflammatory demyelination resulting in neuronal hyperexcitability, axonal swelling, and impaired neuronal function in addition to OL-Kir4.1 loss (colorless channels with dashed borders) and transient upregulation of nodal Kv7 channels. Scale bars: 100 μm (**A** and **F**); 20 μm (**C**–**E**). Original magnification, ×63 (enlarged insets in **D**). *P < 0.05, **P < 0.01, ***P < 0.001, and ****P < 0.0001, by 1-way ANOVA (**C**–**F**).

We next investigated gene expression of potassium voltage-gated channel subfamily Q members 2, 3, and 5 (*Kcnq2/-3/-5*, encoding Kv7.2/-3/-5 channel subunits) in cortical neurons. Analysis of human single-nucleus RNA-Seq (snRNA-Seq) (Figure 2, A and B, and Supplemental Table 2) (12) and mouse single-cell (scRNA-Seq) (42) (Supplemental Figure 4, A and B) cortical data sets revealed high expression levels of *Kcnq2/-3/-5* throughout all human and mouse cortical neuron populations (Figure 2, B and C, and Supplemental Figure 4B), with enhanced *KCNQ3* expression in deep-layer (L4–5) excitatory neurons (ENs) (Figure 2C) (43–45). In MS, we found that *KCNQ3* and *KCNQ5* were downregulated in L2–6 ENs (Figure 2B and Supplemental Table 6), specifically in patients with long disease duration (Figure 2D and Supplemental Table 7). Next, we focused on deep cortical layer neurons in MS and compared *KCNQ3* expression levels between control gray matter (CGM), normal-appearing gray matter (NAGM), and demyelinated gray matter (DMGM) by ISH (Figure 2, E–H). We analyzed brain tissue for the level of demyelination by MOG IR and observed comparable levels of microglial and astroglial reactivity (Supplemental Figure 2A and Supplemental Table 3). Notably, we observed a strong correlation between nuclear and cytoplasmic *KCNQ3* transcripts, emphasizing the validity of snRNA-Seq to assess neuron-specific expression of *KCNQ2/-3/-5* transcripts (Figure 2F and Supplemental Figure 3, A–C). Comparing control with MS, we noted elevated *KCNQ3* transcripts in DMGM neurons compared with CGM, independent of the number of *KCNQ3*-expressing cells (Figure 2G), suggesting an upregulation of *KCNQ3* in cortical deep layer neurons. Then, we found *KCNQ3* expression to be increased in MS tissues from patients with short disease duration, but decreased in those with long disease duration (Figure 2H). We found no correlation with the extent of cortical demyelination, indicating that dysregulation of *KCNQ3* expression was independent of tissue demyelination (Supplemental Figure 3D). We next performed pseudotime trajectory analysis and confirmed a temporal “early” upregulation of *KCNQ2/-3/-5* transcripts and a “late” downregulation, the latter corresponding to chronic stages of inflammation within a MS lesion (Figure 2, I–L, Supplemental Figure 3, E–G, and Supplemental Tables 8 and 9). Similar findings pointing toward reduced *KCNQ3* transcripts in long disease duration were observed in human retinal ganglion cells (RGCs) obtained from MS retina tissue samples (Figure 2, M and N, Supplemental Table 4, and Supplemental Figure 3, H and I). Then, we focused on mouse EAE in early/inflammatory and late/chronic disease stages (Figure 3, A and B, and Supplemental Figure 2B) and assessed neuronal *Kcnq3* and Kv7.2 expression in both cortical layer 4 (L4) neurons, where its expression is most pronounced, similar to humans (compare with Figure 2C), and in RGCs. By quantitative PCR (qPCR) and ISH analysis, we confirmed *Kcnq3* (Figure 3, C and F) but not *Kcnq2/5* (Figure 3E) dysregulation during EAE progression in mouse cortical L4 neurons and RGCs (excitatory projection neurons of the retina) (Figure 3F), but not in non-RGC retinal nuclei (Supplemental Figure 4, C and D). In particular, cortical L4 *Kcnq3* expression gradually increased from the peak of disease (14 dpi) to early-chronic EAE (30 dpi) and then decreased at late-chronic EAE (60 dpi), but did not return to control levels (Figure 3C). We next quantified nodal protein expression of Kv7.2^+^ and found a similar trend of increased Kv7.2 expression during early/inflammatory EAE stages (Figure 3D). Notably, loss of Kv7.2^+^ nodes in late/chronic disease stages confirmed the ISH findings of decreased Kv7 expression, demonstrating RNA-to-protein validation. In parallel, qPCR confirmed dysregulated *Kcnq3* expression in EAE mouse RGCs with transient upregulation in acute/early-chronic disease (Figure 3F).

To summarize, we observed both a loss of OL-KIR4.1 channels and an increase in neuronal *KCNQ3* expression under acute/early inflammatory demyelinating conditions, possibly due to endogenous upregulation to compensate for impaired paranodal K^+^ homeostasis (Figure 3G). However, this mechanism could not be sustained over a prolonged disease duration at both RNA and protein levels (Figure 2, D and H and Figure 3, C, D, and F).

### Mouse EAE is associated with neuronal hyperexcitability during peak disease.

**Figure 4:**
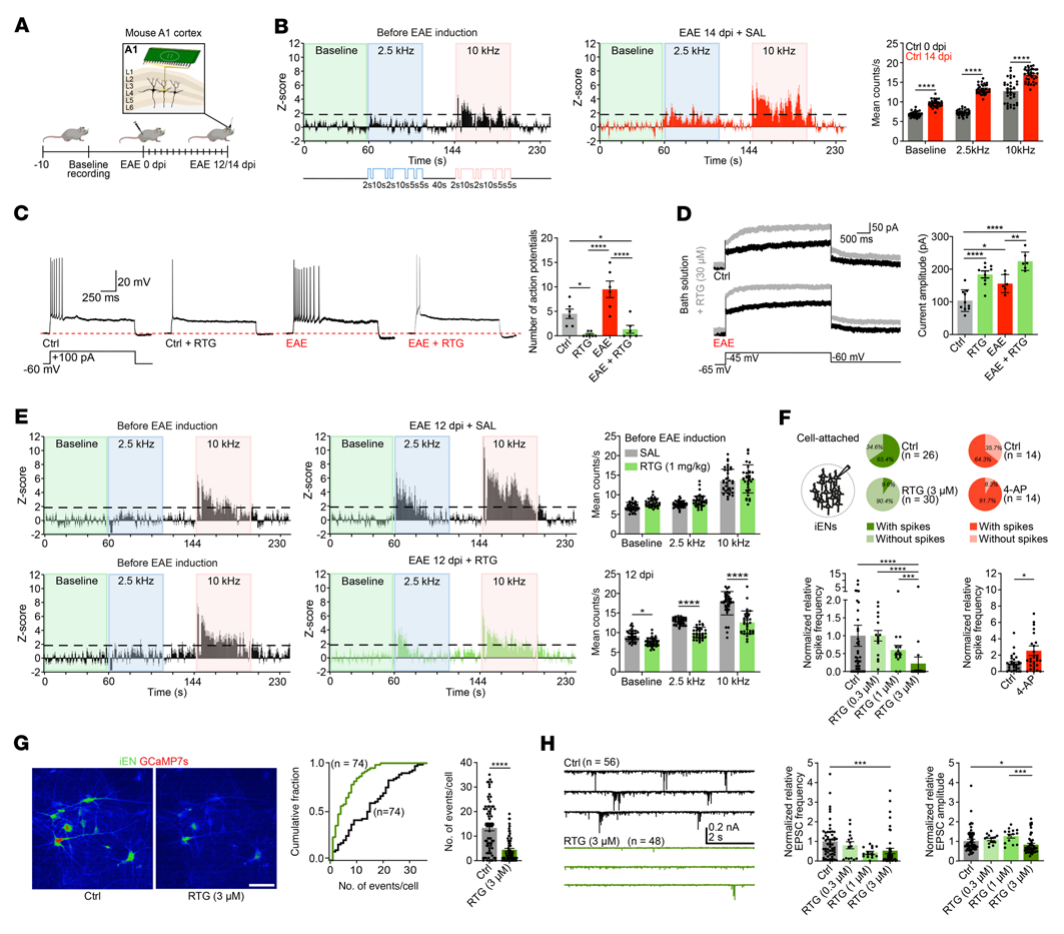
Altered neuronal excitability and network activity in EAE. (**A**) Cartoon illustrates in vivo recordings from mouse A1. (**B**) *Z* score analysis of mouse auditory neurons before EAE induction demonstrated a tonotopic organization of the auditory cortex with a neuronal response to 10 kHz (pink insets) but not 2.5 kHz (blue insets) tones relative to baseline (green insets). EAE induction augmented overall neuronal activity and disrupted tonotopic organization (increased neuronal response to 2.5 kHz) (each, *n* = 34). (**C** and **D**) RTG (30 μM) reduced neuronal excitability (**C**) and increased M-currents (**D**) in control (**C**, untreated, *n* = 6; RTG, *n* = 7; **D**, untreated, *n* = 10; RTG, *n* = 9) and 12 dpi EAE (each *n* = 6) mouse brain sections. (**E**) Continuous RTG treatment (1 mg/kg) starting at baseline prevented an EAE-associated increase in neuronal excitability at 12 dpi (lower panel, right) resulting in similar *z* scores before EAE induction (left panels) (each, *n* = 34). (**F**) RTG (0.3 μM, *n* = 15; 1 μM, *n* = 15; 3 μM, *n* = 30) reduced normalized relative iEN firing in a dose-dependent manner compared with untreated iENs (*n* = 41). Kv channel blocker 4-AP increased spontaneous firing (each, *n* = 29). (**G**) GCaMP7s reporter iENs showed reduced spontaneous calcium transients in response to RTG (3 μM; each, *n* = 74). (**H**) Representative EPSC traces revealed reduced normalized relative EPSC frequency and amplitudes (0.3 μM, *n* = 15; 1 μM, *n* = 14 [amplitude], 15 [frequency]; 3 μM, *n* = 48) of RTG-treated iENs in a dose-dependent manner compared with controls (frequency. *n* = 56; amplitude. *n* = 55). Scale bar: 80 μm (**G**). *P < 0.05, **P < 0.01, ***P < 0.001, and ****P < 0.0001, by 2-way ANOVA (**B**); 1-way ANOVA (**C** and **D**); multiple unpaired *t* tests (**E**); Kruskal-Wallis (left) and Mann-Whitney *U* (right) tests (**F**); 2-way ANOVA (left) and Mann-Whitney *U* (right) (**G**); and Kruskal-Wallis test (**H**).

To characterize central neurophysiological effects under inflammatory demyelinating conditions as a functional correlate of K^+^ dysregulation in MS and EAE, we performed longitudinal recordings of network dynamics and neuronal single-unit activities in A1 areas (Figure 4A). Specifically, we recorded the response to 2 different tones before and after EAE immunization (8, 9, 46) and found enhanced neuronal response and excitability at the peak of disease (14 dpi). Also, 2-tone discrimination at 10 kHz was impaired in EAE (Figure 4B). Furthermore, recordings from L4 primary auditory cortex (A1) neurons (47) confirmed increased firing (Figure 4C), with Kv7-mediated M-currents (Figure 4D) being elevated at the peak of disease (12 dpi).

### Kv7 regulates neuronal excitability under homeostatic and EAE conditions.

To gain more insight into *Kcnq3* function under homeostatic and EAE conditions, we measured L4 neuron excitability in *Kcnq3*-KO animals. As *Kcnq3* and *Kcnq2* subunits mostly form Kv7 channel heterotetramers (48), we first excluded compensatory upregulation of *Kcnq2* in L4 neurons in *Kcnq3-*KO and EAE mice by ISH (Figure 3E). We performed IHC to assess glial cell activation (Iba1, Gfap) and axonal integrity (SMI312) and found no differences between *Kcnq3*-KO and control mice in the SC at different developmental stages (P80 vs. P180) (Supplemental Figure 5, A–C). However, we noted an increase in axonal calibers in older *Kcnq3*-KO animals, suggesting subtle axonal pathologies during aging, reminiscent of OL-*Kcnj10*–cKO mice described previously (49). In addition, shortened AIS lengths were observed in L2/-3 neurons, indicating enhanced levels of neuronal excitability as previously reported (50) (Supplemental Figure 5D). However, loss of *Kcnq3* function had no effects during EAE (Supplemental Figure 5E). Next, we investigated electrophysiological properties and found increased firing of L4 *Kcnq3*-deficient A1 neurons under homeostatic conditions and during EAE (14 dpi) (Supplemental Figure 5, F–H). Also, EAE induction in *Kcnq3*-KO mice further augmented the 2-tone discrimination at 10 kHz (Supplemental Figure 5H).

### Kv7 activation mitigates neuronal hyperexcitability in mouse EAE and reduces excitability in human neurons.

We next explored the effects of pharmacological Kv7 channel opening to balance neuronal hyperexcitability. To assess the effects on neuronal excitability and nodal M-currents, we performed whole-cell recordings (current-clamp and voltage-clamp) under homeostatic and EAE conditions. At the peak of EAE (12 dpi), RTG treatment reduced the number of action potentials in A1 L4 neurons (Figure 4C). Moreover, RTG increased M-currents, in line with its mode of action as a Kv7 channel activator (Figure 4D). Additionally, the increase of M-currents evoked by RTG was more prominent in neurons from EAE mice than in those from non-EAE mice, supporting our previous findings of upregulated Kv7.3 expression at the peak of EAE (Figure 3, C, D, and F, and Figure 4D). As expected, we found that *Kcnq3* deletion led to reduced M-currents with and without RTG (Supplemental Figure 6A). Also, RTG treatment effects were less efficient in both non-EAE and EAE *Kcnq3*-deficient L4 neurons (Supplemental Figure 6B). To assess the effects of RTG on longitudinal in vivo network dynamics, we measured neuronal single-unit activities in A1 areas and observed that RTG-treated EAE mice (Figure 4E) had reduced neuronal hyperactivity (Figure 4B). To clarify whether RTG effects were cell-type specific and could be reproduced in humans, we tested RTG treatment in human pluripotent stem cell–derived (PSC-derived) glutamatergic induced excitatory neurons (iENs) focusing on intrinsic and synaptic properties and spontaneous network activity (Figure 4, F–H). RTG application at various concentrations (0.3 μM, 1 μM, 3 μM) reduced overall iEN firing and decreased spike frequency in a dose-dependent manner, whereas blockade of (voltage-dependent) K^+^ currents with 4-aminopyridine (4-AP) (100 μM) increased spontaneous firing and spike frequency (51) (Figure 4F). RTG treatment in GCaMP7s reporter iENs (3 μM) resulted in reduced spontaneous calcium transients (Figure 4G) and decreased excitatory postsynaptic current (EPSC) amplitudes and frequency, but not EPSC charge (Figure 4H and Supplemental Figure 6E). 4-AP treatment, however, increased EPSC frequency (Supplemental Figure 6F). Whereas RTG (as opposed to 4-AP) did not change the resting membrane potential (Supplemental Figure 6C), it increased the threshold for action potential generation in iENs as compared with 4-AP treatment (Supplemental Figure 6D). To rule out the possibility that 4-AP treatment interfered with Kv7 channel function and Kv7-Kir4.1 K^+^ shuttling, we examined the evoked currents and activity of coexpressed human Kv7.2/Kv7.3 channels in *Xenopus laevis* oocytes and found no effect on these channels, regardless of 4-AP concentration (Supplemental Figure 6G).

### RTG treatment ameliorates EAE symptoms and improves survival.

**Figure 5:**
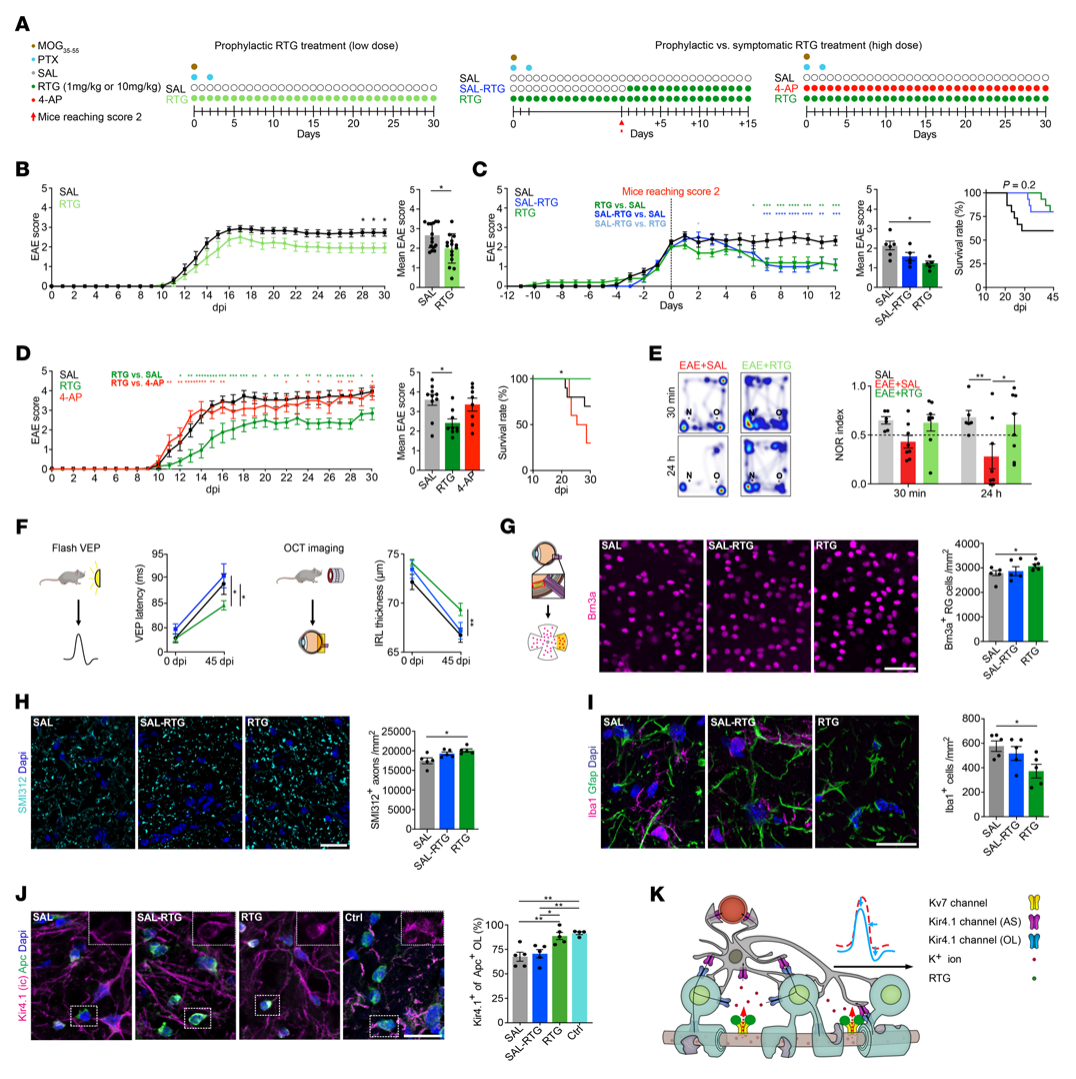
Neuroprotective effects of RTG on structural and functional levels. (**A**) Illustration shows different RTG treatment regimens. (**B**) Prophylactic low-dose RTG treatment (1 mg/kg) attenuated motor deficits in chronic EAE (each *n* = 15). (**C**) Prophylactic and symptomatic (starting at an EAE score ≥2) high-dose RTG treatment (10 mg/kg; both, *n* = 5) attenuated EAE courses compared with SAL-treated controls (*n* = 6). Note that only prophylactic RTG treatment increased survival. (**D**) High-dose RTG treatment (*n* = 9) attenuated early EAE progression and increased survival relative to controls (*n* = 10). 4-AP treatment increased mortality (*n* = 8). (**E**) Memory function decline in SAL-treated EAE was prevented by RTG treatment (each, *n* = 8) compared with non-EAE mice (*n* = 6) in NOR testing 24 hours after habituation (dashed line indicates threshold for memory impairment). (**F**) OCT imaging (45 dpi EAE) showed a thinning of IRLs in SAL-treated mice (*n* = 18) that was prevented by prophylactic (*n* = 22) but not symptomatic (*n* = 9) RTG treatment. VEP latency delay (45 dpi EAE) was improved by prophylactic (*n* = 11) but not symptomatic (*n* = 5) RTG treatment compared with SAL treatment (*n* = 7). (**G**–**I**) Only prophylactic but not symptomatic RTG treatment prevented loss of Brn3a^+^ RGCs (**G**) and SMI312^+^ ON axons (**H**) and decreased Iba1^+^ cell infiltration (**I**) in EAE mice at 45 dpi (each *n* = 5). (**J**) Only prophylactic but not symptomatic RTG treatment prevented OL-Kir4.1 channel loss at 45 dpi in EAE (each *n* = 5), maintaining levels similar to those observed in non-EAE controls (*n* = 4). (**K**) Cartoon illustrates neuron-OL for the K^+^ shuttling mechanism during inflammatory demyelination. Enhanced (transient) neuronal Kv7 channel function augments axonal K^+^ efflux, counteracting neuronal hyperexcitability and contributing to neuroprotection and preservation of OL-Kir4.1 function. Scale bars: 50 μm (**G**); 20 μm (**H**–**J**). *P < 0.05 and **P < 0.01, by mixed-effects analysis with multiple comparisons (left) and, Mann-Whitney *U* test (right) (**B**); 2-way ANOVA (left), Kruskal-Wallis (middle), Mantel-Cox (right) (**C** and **D**); 2-way ANOVA (**E** and **F**); 1-way ANOVA (**G**–**J**).

We next tested the in vivo effects of chronic RTG treatment on EAE symptoms, functional readouts, and neuroglial pathology in 3 different treatment groups (Figure 5A). Prophylactic low-dose (1 mg/kg) RTG treatment only attenuated disease severity during chronic disease (28–30 dpi) (Figure 5B), whereas high-dose (10 mg/kg) prophylactic RTG treatment also ameliorated EAE progression at the onset of clinical symptoms (12–14 dpi) and resulted in higher survival rates (Figure 5, C and D). Furthermore, both prophylactic and symptomatic high-dose RTG treatment attenuated disease symptoms in chronic EAE, whereas only prophylactic high-dose RTG treatment remained significant in the mean EAE score and increased survival (Figure 5C). To assess overall axonal damage in EAE and RTG treatment effects, we measured the serum neurofilament light chain (sNfL) levels (52) that correlated with the EAE scores for saline- (SAL-) and RTG-treated EAE mice at 14 dpi and 28 dpi, corresponding to peak and chronic disease states (Supplemental Figure 7C). Notably, the SAL-treated mice showed increased EAE scores and sNfL levels compared with RTG-treated animals. In contrast, nonspecific K^+^ channel blocking with 4-AP (see Figure 4) failed to alleviate EAE symptoms and resulted in increased mortality at 30 dpi (Figure 5D). Also, 4-AP treatment led to increased ON axon calibers, indicating subtle axon pathology (Supplemental Figure 7A), but had no effect on inflammation (Supplemental Figure 7B).

### RTG treatment improves optic pathway function and limits neuroinflammation.

We used the novel object recognition (NOR) test to assess recognition memory skills in EAE mice, which revealed an impairment in long-term memory (24 h after habituation) upon EAE induction. This impairment was prevented by prophylactic RTG treatment starting from the day of EAE induction (Figure 5E and Supplemental Figure 7D). To investigate function of the anterior visual system in EAE, we recorded flash-light visual evoked potentials (VEPs) (53, 54). Notably, we found that only high-dose prophylactic, but not symptomatic, RTG treatment was effective in reducing EAE-related delays of VEP latencies and thus prevented ON dysfunction at 45 dpi (Figure 5F). Next, we examined retinal layer integrity during EAE by optical coherence tomography (OCT). We observed EAE-related thinning of inner retinal layers (IRLs) reflecting retinal neurodegeneration (55), which was ameliorated by prophylactic RTG treatment. Prophylactic RTG treatment demonstrated protective effects against EAE-related neuronal loss in the visual system by preserving RGCs and SMI312^+^ axon density in ON tissue 45 dpi (Figure 5, G and H). Also, prophylactic RTG treatment resulted in ameliorated inflammatory activity relative to SAL treatment (Figure 5I). Consistent with findings in MS ON (Figure 1G), OL-Kir4.1 channel expression was decreased in EAE ONs compared with non-EAE control ONs; however, prophylactic RTG treatment maintained OL-Kir4.1 expression at preimmunization baseline levels (Figure 5J). To rule out immune-related effects of RTG treatment, we profiled brain-infiltrating immune cells and observed no differences between SAL- and RTG-treated animals (Supplemental Figure 7E).

In conclusion, prophylactic RTG treatment was well tolerated, improved functional and structural EAE outcomes, and delayed neuronal loss in the absence of an antiinflammatory effect (Figure 5K).

### Chronic RTG treatment is beneficial in mice with OL-Kcnj10 loss of function.

**Figure 6:**
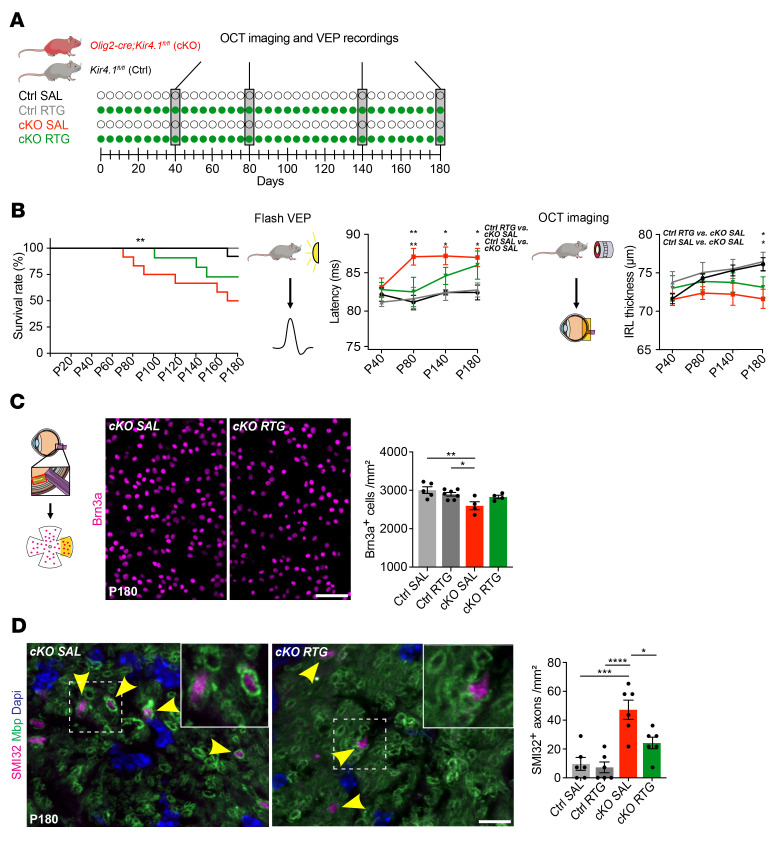
Effects of chronic RTG treatment in OL-*Kcnj10*–deficient mice. (**A**) Scheme of continuous RTG versus SAL treatment in OL-*Kcnj10*–KO mice versus controls. (**B**) Chronic RTG treatment increased survival in both control (SAL, *n* = 13; RTG, *n* = 12) and OL-*Kcnj10*–KO (SAL, *n* = 12; RTG, *n* = 11) animals at P180. Delayed VEP latencies in SAL-treated but not RTG-treated (both *n* = 5) OL-*Kcnj10*–KO mice versus SAL-treated (*n* = 9) and RTG-treated (*n* = 8) mice at P80. Note the delayed VEP latencies with aging in both SAL- and RTG-treated KO groups at P140 and P180. IRLs showed physiological growth during aging in both SAL- and RTG-treated (both, *n* = 8) control mice and RTG-treated but not SAL-treated (both *n* = 5) OL-*Kcnj10*–KO animals until P140. At P180, IRL thinning occurred only in SAL-treated but not RTG-treated KO mice. (**C**) Chronic RTG treatment (*n* = 4) prevented loss of Brn3a^+^ RGCs in SAL-treated (*n* = 5) OL-*Kcnj10*–KO mice compared with SAL-treated (*n* = 5) and RTG-treated (*n* = 7) controls. (**D**) RTG treatment prevented increased counts of dystrophic/damaged SMI32^+^ axons in the ON, as seen in SAL-treated OL-*Kcnj10*–KO mice compared with controls (*n* = 6 for each group). Yellow arrowheads indicate SMI32^+^ (dystrophic) axons sheathed by Mbp^+^ myelin. Scale bars: 20 μm (**C**); 10 μm (**D**). Original magnification, ×63 (enlarged insets in **D**). *P < 0.05, **P < 0.01, ***P < 0.001, and ****P < 0.0001, by Mantel-Cox (left), 2-way ANOVA (right, VEP and OCT) (**B**); 1-way ANOVA (**C** and **D**).

We previously showed that loss of OL-*Kcnj10* function results in progressive neurodegeneration, thus resembling aspects of chronic EAE and progressive MS (49). To investigate whether pharmacological Kv7 opening might also be beneficial when para- and juxtanodal K^+^ siphoning through OL cells is impaired, we tested OL-*Kcnj10*–KO mice and control littermates with high-dose RTG (10 mg/kg daily) compared with SAL over 5 months, starting at P40, until 6 months of age, corresponding to P180 (Figure 6A). Chronic RTG treatment improved VEP latencies at P80, but not at later time points; likewise, RTG treatment could assuage but not prevent age-related IRL thinning (Figure 6B). Nevertheless, RTG treatment resulted in increased survival of OL-*Kcnj10*–KO mice (Figure 6B). At the structural level, RTG protected RGCs from degeneration in OL-*Kcnj10*–KO mice and showed protective effects on the survival of SMI312^+^ axons and CASPR^+^ paranodes (Figure 6C and Supplemental Figure 8, A and B). RTG treatment also protected against an accumulation of dystrophic SMI32^+^ axons and an increase in axon caliber at P180 in OL-*Kcnj10*–KO mice (Figure 6D and Supplemental Figure 8A). In addition, we noted a mild reduction of inflammation but no effects on astrogliosis in RTG-treated OL-*Kcnj10*–KO ONs at P180 (Supplemental Figure 8C).

## Discussion

We describe a “nodal-glial” interface that appeared to be necessary for long-term neuron-OL K^+^ shuttling and homeostasis during inflammatory demyelination and demonstrate the importance of nodal-glial interaction during health and disease, similar to previous work on neuron-microglia crosstalk (56). This spatial and functional interface comprises outward-rectifying Kv7 (41) and inward-rectifying OL-Kir4.1 K^+^ channels at the NoR and the AIS (49). During repolarization, Kv7 channels shuttled K^+^ ions from the axolemma into paranodal spaces, where K^+^ was “siphoned” to areas of low K^+^ concentration by astroglial and OL-Kir4.1 channels. While both neuronal and OL channels were dysregulated in human MS and mouse EAE, OL-Kir4.1 channels became chronically downregulated in WM MS and EAE tissues. In contrast, Kv7 channel subunits (in particular Kv7.3) appeared to be transiently upregulated during early inflammatory periods and downregulated in chronic disease.

Our findings of Kv7 channel upregulation in acute disease stages are consistent with other work on altered expression and redistribution of sodium channels along demyelinated axons in MS and related models (57, 58), resulting in conduction defects, breakdown of ion gradients, and axonal degeneration (6, 59). Moreover, shortened internodal length and an increase in nodal density with a concomitant increase in sodium channels are associated with enhanced axonal/neuronal excitability (59, 60), thus providing an explanation for increased Kv7 expression not only due to an increase in nodal density but also as an adaptive response to hyperexcitability.

Might early and transient upregulation of Kv7 subunits represent an endogenous compensatory mechanism that counteracts enhanced neuronal excitability in MS and EAE? If so, our data demonstrate that this mechanism ultimately failed during progressive disease characterized by a combined loss of Kir4.1 and Kv7 channel function. Specifically, we found that impaired Kv7 function was driven by dysregulated *Kcnq3* (but not *Kcnq2*) gene expression during EAE. We further demonstrated that chronic *Kcnq3* loss of function increased neuronal excitability ex vivo and in vivo, pointing toward a key role for this subunit in network function under physiological and disease conditions. However, whereas lost *Kcnq3* function did not worsen EAE, we found that chronic loss of OL-*Kcnj10* function worsened symptoms and increased mortality in EAE. Such loss-of-function studies thus provide evidence that Kv7 and Kir4.1 channels function in a synergistic way to establish and maintain saltatory conduction over time. Eventually, this synergistic interplay collapses under chronic inflammatory demyelinating conditions such as those in progressive MS and EAE.

Finally, we tested whether it was possible to stabilize K^+^ homeostasis and prevent neuronal hyperexcitability during EAE using small molecules that could potentially translate to the clinic. To activate outward-rectifying currents at the NoR, we treated EAE mice with RTG, a specific Kv7 channel opener. Through ex vivo recordings, we validated that RTG treatment fostered axonal K^+^ outflow and reduced the firing rate of neurons in both control and EAE brain tissues. Under in vivo conditions, we found that RTG treatment starting at EAE induction improved neurological and cognitive symptoms and prevented neurodegeneration in EAE and during early adulthood in cKO animals lacking OL-Kir4.1 channels. We demonstrated that these effects were consistent between various functional circuits comprising the visual, auditory, and motor systems. Also, RTG treatment mitigated the loss of OL-Kir4.1 channel expression in EAE, probably because of overall improved tissue preservation and increased axonal K^+^ outflux stimulating OL-mediated siphoning of K^+^ ions. Collectively, our data suggest that “early” neuroprotection through the Kv7 channel opening might represent an interesting approach to delay clinical progression and “late” neurodegeneration by stabilizing neuron-OL K^+^ shuttling over a prolonged period.

Unlike with RTG treatment, clinical symptoms were not altered and mortality was increased in EAE mice treated with 4-AP, a fairly nonspecific Kv channel blocker with no obvious effects on Kv7 channel function, as demonstrated in our study. Of note, our data refute previous results in which EAE animals were treated with 4-AP via the drinking water versus the daily i.p. injections in our study (61), which, given the different routes of administration, makes a direct comparison difficult. The increase in mortality was most likely the consequence of neuronal hyperexcitability and the development of epileptic seizures, which were regularly observed in EAE animals treated with 4-AP. These opposing effects could be validated in vitro when recording from human iEN cells treated with either RTG or 4-AP. Here, we found that RTG, but not 4-AP, could reduce neuronal firing, increased the threshold for action potential generation, and, moreover, was able to stabilize neuronal network activity. Notably, we cannot completely rule out antiinflammatory effects of RTG treatment, although our data rather suggest a primary role in regulating neuronal excitability with potentially secondary antiinflammatory effects due to improved neuronal preservation.

Previously, the most common adverse effects of RTG treatment in humans were dose related and included somnolence and dizziness, whereas long-term therapy was associated with skin and retina discoloration. Rare but potentially serious adverse effects of previous formulations included urinary retention and a reduction in visual acuity due to retinal pigmentation. Hence, new drug formulations targeting Kv7 channels need to be evaluated, particularly in the context of visual impairment during long-term treatment (38–40, 62, 63).

In summary, our findings highlight neuron-OL compensatory mechanisms in chronic inflammatory demyelination and suggest a rational neuroprotective approach through modulation of K^+^ levels. Treatment with RTG provided proof of concept for this notion, showing how endogenous Kv7 might be recruited to counteract neuronal hyperexcitability in progressive MS and related neuroimmune diseases. Thus, pharmacological Kv7 channel activation represents a promising therapeutic strategy to counteract progressive neurodegeneration in MS and beyond.

## Methods

### Transgenic mouse lines.

*Kcnj10*-cKO mouse strains were maintained at the UCSF in a specific pathogen–free animal facility. cKO mice were obtained by crossing *Kir4.1^fl/fl^* mice with *Cnp-Cre* mice. *Cnp-Cre*–transgenic mice were generated in-house and had been previously generated (64). *Kir4.1^fl/fl^* mice were obtained from Ken D. McCarthy (UNC School of Medicine, Chapel Hill, North Carolina, USA) and had been generated as previously described (22, 65). *Olig2-tva-Cre*–transgenic mice were generated as previously described (66). Long-term treatment experiments (Figure 6 and Supplemental Figure 8) were performed with *Olig2-Cre* *Kir4.1^fl/fl^* and Cre-negative *Kir4.1^fl/fl^* mice. *Kcnq3*-KO mouse strains were maintained at the animal facility of the UKM under specific pathogen–free conditions. *Kcnq3^–/–^* mouse strains were generated in-house and have been previously described (67, 68). All mice were maintained on a C57BL/6J background. *Kir4.1^fl/fl^* littermates and WT mice were used as controls.

### Mouse tissue processing.

Mice were transcardially perfused with PBS or 4% paraformaldehyde (PFA) for IHC and 4% formaldehyde and 0.2% glutaraldehyde in 0.1 M PBS containing 0.5% NaCl for immunolabeling. For IHC analysis, cryosections of mouse and human (snap-frozen) tissues were collected using a CM3050S cryostat (Leica Microsystems). For EM analysis, tissue was postfixed for 24 hours, embedded in 10% gelatin, and infiltrated overnight with 2.3 M sucrose in 0.1 M PBS. Ultrathin cryosections of embedded tissue in gelatin blocks were made using a UC7 Cryo-Ultramicrotome (Leica) and a 35° Cryo Immuno Diamond knife (Diatome).

### Conventional IHC.

Sections were fixed in ice-cold methanol or 4% PFA and blocked in PBS-T and 10% goat serum (1% BSA, 0.2% fish skin gelatin, and 0.1% Triton in 0.1 M PBS for βIV-spectrin staining) for 30 minutes (at least 60 min for βIV-spectrin staining). Primary antibody incubations were carried out overnight at 4°C, and slides were incubated with secondary antibodies for 2 hours. For chromogenic assays, sections were incubated with biotinylated secondary IgG antibodies (Thermo Fisher Scientific, catalog 62-6540, goat anti–mouse IgG [H+L] cross-adsorbed secondary antibody, biotin; 1:500) followed by an avidin-biotin complex for a 1-hour incubation (Vector Laboratories, 1:500) and subsequent color revelation using diaminobenzidine (Dako). H&E staining was carried out for diagnostic purposes. Slides with fluorescent antibodies were mounted with DAPI Fluoromount-G (Thermo Fisher Scientific).

### Primary antibodies.

The following antibodies for IHC and Western blotting were used: mouse anti-CASPR (75-001, NeuroMab, 1:250); rabbit anti-CASPR (ab34151, Abcam, 1:1,000); rabbit anti-Kv7.2 (ab22897, Abcam, 1:250); rabbit anti-Kv7.2 (368103, Synaptic Systems, 1:300 to 1:1,000); guinea pig anti-Kv7.2 (raised against peptide VQKSRNGGVYPGTSGEKKL, coupled by a C-terminally added cysteine to a keyhole limpet hemocyanin named Q2Agp1 (67) (generated in-house; 1:500); rat anti–human KIR4.1 (clone 20F9 generated against an extracellular domain of human KIR4.1 [peptide sequence AHGDLLELDPPANHT], 1:1,000; generated in-house); rabbit anti-KIR4.1 (APC-165, Alomone Labs, 1:1,000); rabbit anti-KIR4.1 (APC-035, Alomone Labs, 1:3,000); mouse anti–NOGO-A (clone 11C7, gift from Martin Schwab, University of Zurich, Zurich, Switzerland; 1:3,000); mouse anti-MOG (clone 8-18C5, Millipore Sigma, 1:1,000 [1:200 after ISH]); rat anti-MBP (MAB386, clone 12, Merck, 1:200); rabbit anti-Iba1 (019-19741, Wako, 1:500); mouse anti-SMI312 (clone SMI312, 837904, BioLegend, 1:1,000); anti-GFAP (13-0300, Thermo Fisher Scientific, 1:200); rabbit anti–βIV-spectrin (provided by Maren Engelhardt, University of Linz, Linz, Austria, 1:1,000) (50); chicken anti-NeuN (ab134014, Abcam, 1:1,000); goat anti-Brn3a (sc-31984, Santa Cruz Biotechnology, 1:200); mouse anti–neurofilament H (NF-H), nonphosphorylated (clone SMI32, 801701, BioLegend, 1:10,000); and anti-APC (clone CC1, OP80, MilliporeSigma, 1:500). For all experiments, negative control sections without primary antibodies were processed in parallel. All IHC analyses were carried out in a blinded manner.

### RNA ISH.

Chromogenic single-molecule and multiplex ISH was performed according to published protocols (12, 69) and the manufacturer’s recommendations (RNAscope 2.5 HD Reagent Kit-RED; RNAscope Multiplex Fluorescent v2 Assay Kit, ACD Biotechne). The following manual RNAscope assay probes were used: *KCNQ3* (human, chromogenic single ISH); *KCNJ10*, *SYT1*, and *PLP1* (human, multiplex ISH); and *Kcnq3*, *Kcnq2*, *Syt1*, *Rorb*, *Plp1*, and *Kcnj10* (mouse, multiplex ISH). For multiplex ISH, probes were labeled with TSA Plus Fluorophores (Fluorescein, Cyanine3, Cyanine5, Akoya Biosciences), and nuclei were labeled with DAPI. Following red chromogenic single-molecule ISH, IHC and hematoxylin staining of nuclei were performed. For quality control, negative (DapB) and positive ISH probes (*PPIB*, *Polr2a*, *Ppib*, and *Ubc*) were run in parallel.

### Quantification of in situ RNA transcripts.

The number of individual RNA signals was quantified using the automated particle analysis plug-in for ImageJ Fiji software, version 2.1 (NIH). For human *KCNQ3* quantification, 11 MS samples were screened for demyelination using MOG IHC. Seven NAGM and 20 DMGM upper cortical layer (L1–3) and 20 NAGM and 14 DMGM deep cortical layer (L4–6) areas were selected. For 6 control samples, 19 upper layer and 18 deep layer areas were selected. Three to 4 regions of interest (ROIs) were quantified for each area, and the mean value per condition and layer was assessed to determine the average *KCNQ3* expression per sample.

### Human and mouse snRNA-Seq data acquisition for bioinformatics analyses.

Expression matrices and associated metadata were obtained from the UCSC Cell Browser (https://cells.ucsc.edu/?ds=ms) for the Schirmer et al. data set (12) (PRJNA726991) and the NCBI’s Gene Expression Omnibus (GEO) database for the Tasic et al. data (42) (GEO GSE115746). For analysis of the Schirmer et al. data set (12), the expression matrix was normalized using Seurat (version 3.2.3) SCTransform (70) with default parameters. Principal component analysis (PCA) was done using Seurat RunPCA() calculating top 50 principal components (PCs). Fifteen PCs were taken as input for Seurat RunUMAP(), FindNeighbors(), and FindClusters(). Finally, a subset of neurons and samples originating from the London Brain Bank (Supplemental Table 5) were renormalized using the same procedure and included 14 PCs for clustering. The Tasic et al. data set (42) was reanalyzed in the same way. Since barcode annotation discrepancies were found between matrix and metadata provided by the authors, only the barcodes present in both the matrix and metadata were included (Supplemental Table 10).

### Trajectory inference analysis.

A trajectory for human excitatory cortical L2/-3 neurons was inferred using the Monocle package, version 3 beta (71). Briefly, transcriptomes of all L2/-3 nuclei were dimensionally reduced by computing 50 PCs; batch effects were removed using the batchelor algorithm (72), where each sample was treated as a batch. Next, an unsupervised trajectory was inferred through the reduced space. The resulting tree was ordered with the tree root where most nuclei of the control samples clustered. Finally, branches were analyzed separately by dividing branches between the starting point (root) and the ending point (furthest point of the pseudotime trajectory).

### Human induced PSC–derived neurons.

Induced excitatory forebrain neurons (iENs) were differentiated from induced pluripotent stem cells (iPSCs) derived from a healthy donor (HD6, Heidelberg University, Heidelberg, Germany) according to a protocol developed by Zhang et al. (73). iPSCs were infected with lentiviruses expressing rtTA (ubiquitin promoter), neurogenin 2 (NGN2) (73), and puromycin (rtTA promoter). After 1 day, doxycycline was used to trigger NGN2 and puromycin expression, followed by puromycin selection for 2 days. For calcium imaging, cells were additionally infected with a lentivirus expressing GCaMP7s (ubiquitin promoter). All measurements were performed 4–5 weeks after derivation. Coverslips containing iENs were placed in a recording chamber under an Olympus BX51WI microscope equipped with differential interference contrast (DIC) and fluorescence capabilities. Recordings were performed at 24°C ± 1°C. In all experiments, 4-AP (MilliporeSigma; 100 μM) and RTG (Alomone; 0.3 μM, 1 μM, 3 μM) were bath-applied. A CMOS camera (Thorlabs) was used to image in green channel (100 ms pulses, LED 488 nm) every 2 seconds. In the patch-clamp experiments, cells were approached and patched under DIC with 3 MΩ pipettes using a Narishige PC-10 puller. Neurons were maintained at –70 mV holding potentials using an Axoclamp 700B amplifier controlled by Clampex11 software (Molecular Devices). Series resistance varied between 8 and 10 MΩ. iENs in the recording chamber were continuously perfused with oxygenated (95% O_2_ / 5% CO_2_) bath solution containing 125 mM NaCl, 2.5 mM KCl, 1 mM MgCl_2_, 2 mM CaCl_2_, 25 mM glucose, 1.25 mM NaH_2_PO_4_, 0.4 mM ascorbic acid, 3 mM myoinositol, 2 mM Na-pyruvate, and 25 mM NaHCO_3_ (pH 7.4, 315 mOsm). Synaptic currents were recorded with an internal solution that contained 140 mM Cs-methanesulfonate, 0.5 mM EGTA, 1 mM MgCl_2_, 10 mM HEPES, 2 mM ATP-magnesium, 0.4 mM GTP-sodium, and 10 mM Na-phospho-creatine, (pH 7.2, 310 mOsm). Intrinsic and action potential properties were recorded using a pipette solution containing 125 mM KMeSO_3_, 20 mM KCl, 2 mM MgCl_2_, 0.5 mM EGTA, 4 mM MgATP, 0.3 mM NaGTP, 10 mM Na-phosphocreatine, and 10 mM HEPES-KOH (pH adjusted to 7.3, 315 mOsm). Current-clamping membrane potentials were kept at approximately –70 mV using small (<30 pA) holding currents, and step currents were injected to elicit APs. Analysis and plotting of imaging data were performed using FluoroSNNAP (https://www.seas.upenn.edu/~molneuro/FluoroSNNAP/user_guide.pdf) and custom R macros (https://github.com/AcunaLabUHD/Physiology-Macros/blob/main/calcium%20summaries.v1.1.R; committ ID: 8f13ac5. Spikes and synaptic current properties were analyzed using custom macros written in IgorPro 6.11 (Wavemetrics) (https://github.com/AcunaLabUHD/Physiology-Macros/blob/main/synaptic%20transmission_v4.3.ipf; commit ID: 8f13ac5) or Clampfit11 software (Molecular Devices). Data derived from different batches were normalized to the control condition.

### Tissue preparation for electrophysiological experiments.

Naive and MOG_35−55_-immunized C57BL/6J and *Kcnq3-*KO female mice (8–12 weeks old) were anesthetized and decapitated. Brains were removed and dissected in ice-cold slicing solution containing 2.5 mM KCl, 1.25 mM NaH_2_PO_4_, 10 mM MgSO_4_, 20 mM PIPES, 10 mM glucose, 200 mM saccharose, and 0.5 mM CaCl_2_ , pH 7.35. Acute coronal brain slices containing the primary auditory cortex (A1) were prepared on a vibratome (Leica) and then incubated for 30 minutes in a warm (32°C) carbogenated solution containing 1.25 mM NaCl, 240 mM NaHCO_3_, 12.5 mM NaH_2_PO_4_, 25 mM KCl, 10 mM glucose, 2 mM CaCl_2_, and 2 mM MgSO_4_, pH 7.35.

### Whole-cell patch-clamp recordings.

Following an incubation period of 30 minutes at room temperature, slices were transferred to a recording chamber constantly perfused at a flow rate of approximately 2 mL/min with carbogenated artificial cerebrospinal fluid containing 120 mM NaCl, 2.5 mM KCl, 1.25 mM NaH_2_PO_4_, 22 mM NaHCO_3_, 25 mM glucose, 2 mM CaCl_2_, and 2 mM MgSO_4_, pH 7.35. Whole-cell recordings were performed on the soma of L4 A1 pyramidal neurons using glass patch pipettes filled with a K^+^ gluconate–based intracellular solution containing 10 mM NaCl, 88 mM K-gluconate, 20 mM K_3_-citrate, 10 mM HEPES, 3 mM BAPTA, 15 mM phosphocreatine, 1 mM MgCl_2_, 0.5 mM CaCl_2_, 3 mM Mg-ATP, 0.5 mM Na-GTP, pH 7.25 (295 mOsmol/kg). Typical electrode resistance was 5–6 MΩ. Series resistance was within 5–15 MΩ, and a compensation of 40% or greater was routinely applied. Patch pipettes were connected to an EPC-10 amplifier (HEKA Elektronik) to measure electrical activity. Recordings were governed by Patchmaster software (HEKA Elektronik) and corrected for the liquid junction potential.

### Current-clamp analysis.

Current-clamp experiments were performed in a bath solution without blockers (see above). The resting membrane potential was adjusted to –60 mV by DC current injection, and the degree of single-cell excitability was characterized in response to a depolarizing current injection of 100 pA (1.5 s). The number of action potentials was assessed under control conditions and following application of RTG (30 μM) to the bath solution. FitMaster (HEKA Elektronik) and PEAK Software were used for the analysis (Meuth IT Consulting).

### Voltage-clamp recordings.

Variations in current amplitude were measured in a bath solution (see above) containing 2 μM mibefradil, 1 μM nifedipine, 30 μM ZD7288, and 0.5 μM tetrodotoxin. Membrane outward currents were elicited at a holding potential of –65 mV, followed by a depolarizing step to –45 mV. Thereafter, neurons were repolarized to –60 mV. The duration of each pulse was 4 seconds. Current amplitudes were analyzed at the furthermost 500 ms of the depolarizing voltage step of –45 mV under control conditions and following RTG application (30 μM) using FitMaster software (HEKA Elektronik).

### Stereotactic surgeries.

For craniotomy surgery and electrode implantation, anesthesia was administered and maintained (8). Animals were anesthetized and positioned in a stereotactic frame (ASI Instruments). The skin was incised, the skull was disinfected and leveled, and a craniotomy was performed (A1, anteroposterior, –2.18 mm; lateral, +4.2 mm from bregma; dorso-ventral, 1 mm from brain surface). Microwire arrays housing 8 electrodes (Stablohm 650, California Fine Wire) were implanted unilaterally (left hemisphere) into cortical L4 of A1 and fixed with dental cement (GlassLute, PULPDENT). A ground electrode was placed in proximity of the midline over the cerebellar region (anteroposterior, –5.8; lateral, +0.5 mm from bregma, right hemisphere). Gold-plated wire tips with a cathodal current of 1 μA were submerged in a gold solution to reduce the impedance to 150–300 kΩ. The animals’ health status and recovery were assessed daily for 10 days after the procedure.

### In vivo electrophysiological recordings.

After 10 days of recovery following surgery, C57BL/6J and *Kcnq3*-KO mice were MOG_35−55_ immunized, and recordings of unit activity were performed at 12 and 14 dpi. Recordings obtained prior to EAE induction served as the control. Recordings were performed in freely moving mice before and during the presentation of an auditory stimulus consisting of 6 repetitive low- or high-frequency tones (2.5 kHz, 10 kHz at 85 dB). Extracellular unit activities were recorded using a Multichannel Amplifier System (Alpha Omega) and stored on a personal computer. Signals were band-pass filtered at 100 Hz to 20 kHz and processed at a sampling rate of 40 kHz. Individual neuronal spikes were sorted by time amplitude window discrimination and PC analysis (Offline Sorter, Plexon) and validated through quantification of cluster separation (9).

### Single-unit analysis.

Firing rates and *z* scores of sorted neurons were analyzed relative to baseline activity in 1 second segments (1 bin), 60 seconds before and during the presentation of an auditory stimulus, with a custom MATLAB interface (MathWorks). Firing rates of individual units were *z* scored to their mean baseline activity. Neurons were considered responsive if 1 or more bins showed a *z* score of 1.96 or higher (*P* = 0.05) in response to stimulus presentation (9, 46).

### EAE induction and clinical scoring.

EAE was induced in female C57BL/6J mice (The Jackson Laboratory) at 8–14 weeks of age as previously described (74). Mice were immunized s.c. with MOG_35–55_ peptide (100 μg, Anaspec, UCSF; 200 μg, Peptides & Elephants, UKE; 200 μg, Charité, UKM) in complete Freund’s adjuvant (CFA) containing nonviable *Mycobacterium tuberculosis* (1 mg/mL, UKE; 2 mg/mL, UCSF; 5 mg/mL, UKM; Difco). In addition, pertussis toxin (200 ng, List Biological Laboratories, UCSF; 300 ng, Calbiochem, UKE; 400 ng, Enzo Life Sciences, UKM) was injected i.p. on the day of immunization (0 dpi) and 48 hours later (2 dpi). Mice were scored daily: 0 = no clinical signs; 0.5 = distal limp tail; 1 = limp tail; 1.5 = inability to turn immediately when flipped on the back; 2 = weakness of hind limb; 2.5 = severe hind limb paresis; 3 = severe bilateral hind limb paresis with paralysis of 1 hind limb; 3.5 = complete bilateral hind limb paralysis; 4 = beginning forelimb paresis; 4.5 = severe forelimb paresis; 5 = moribund. Animals reaching a clinical score of either 3.5 or higher for more than 4 (UKM) or 7 (UKE) consecutive days or a clinical score of 4 or higher (UKM, UKE, UCSF) were removed from experimentation and euthanized. The last score observed was included for the analysis until the last experimental time point. Cumulative clinical scores were determined as the mean scores for each mouse from the day of clinical onset and for the total duration of the experiments, divided by the number of days of sickness.

### RTG and 4-AP treatment in EAE mice.

Mice from treatment groups received either RTG, 4-AP, or SAL as i.p. injections. RTG (1 mg/kg or 10 mg/kg body weight) and 4-AP (250 μg/kg) were given as a sterile solution dissolved in SAL, as described in other reports (75). To distinguish presymptomatic and symptomatic treatment, EAE mice were treated daily starting at 0 dpi with either SAL, RTG, or SAL with a change to RTG after achieving an EAE score of 0.5 or higher (1 mg/kg BW) or 2 or higher (10 mg/kg BW). For chronic RTG treatment of transgenic mice, RTG (10 mg/kg) was applied between P40 and P180 according to previous reports (76–78). Control mice were injected i.p. daily with the same volume of 0.9% SAL.

### Data and material availability.

Human snRNA-Seq data were obtained from the UCSC Cell Browser (https://cells.ucsc.edu/?ds=ms; accession number PRJNA726991). Mouse snRNA-Seq data were obtained from the NCBI’s Gene Expression Omnibus (GEO) database (GEO GSE115746).

### Statistics.

All results are presented as the mean ± SEM. Statistical analysis was performed using parametric (unpaired *t* test comparing 2 groups; 1-way or 2-way ANOVA comparing 3 or more groups) or nonparametric (Kruskal-Wallis test comparing 3 groups) tests. Tests were performed using 2-tailed analysis unless stated otherwise. The significance cutoff was set at a *P* value of less than 0.05. Statistical analyses were performed using GraphPad Prism 9.0 (GraphPad Software).

### Study approval.

Human postmortem tissue samples were obtained from the UK MS Society Tissue Bank (London, United Kingdom), the Netherlands Brain Bank (Amsterdam, Netherlands), and Johns Hopkins University Medical School (Baltimore, Maryland, USA) following fully informed consent by tissue donors according to national ethics guidelines and legal regulations. In particular, ethics approval was obtained from the UK National Research Ethics Committee (08/MRE09/31) and the Independent Review Board of the Vrije Universiteit Medical Center Amsterdam (IRB00002991, 2009/148). Control tissue samples were obtained from individuals without neuropathological findings. In total, postmortem brain tissue blocks from 10 patients with MS and 6 controls, ON tissues from 9 patients with MS and 4 control individuals, and eyeball/retinal tissues from 7 patients with MS and 6 controls were used. Supplemental Tables 1–4 provide details on the human tissue samples. Animal experiments were performed at the animal facilities of UKM, UKE, and UCSF in accordance with national ethics guidelines and legal regulations (Landesamt für Natur, Umwelt und Verbraucherschutz Nordrhein-Westfalen, approval ID 81-02.04.2018.A266/84-02.04.2015.A585 [UKM]; Behörde für Soziales, Familie, Gesundheit und Verbraucherschutz Hamburg,G122/17 [UKE]; IACUC and Laboratory Animal Resource Center San Francisco, AN110094 [UCSF]). All mice were maintained on a 12-hour light/12-hour dark cycle with food and water available ad libitum.

## Authors contribution

HK, LF, JD, DHR, SGM, and LS designed the experiments. HK, LF, JD, SS, ACH, JC, CM, ED, WM, CC, AKP, MG, AZ, VNN, AD, M Cerina, TM, CLM, JKS, JHS, PD, NR, KS, M Chavali, RS, KCF, and LS performed the experiments. HK, LF, JD, SS, CM, AZ, PD, and LS analyzed the data. HK and LS wrote the manuscript. CS, BH, GS, MP, TJJ, ME, TB, KAN, PAC, MAF, AJG, CA, DHR, SGM, and LS participated in data analysis and discussion of results. HK and LS completed the final review and submitted the manuscript. All authors contributed to the article and approved the submitted version. The order of the co–first authors was determined on the basis of their efforts and contributions to the study.

## Supplementary Material

Supplemental data

Supplemental tables 1-10

## Version 1 01/31/2023

In-Press Preview

## Version 2 04/03/2023

Electronic publication
