# Supplementary material for: Neuron-oligodendrocyte potassium shuttling at nodes of Ranvier protects against inflammatory demyelination
Source: J Clin Invest. 2023 Apr 3;133(7):e164223. doi: 10.1172/JCI164223 (PMC10065072; doi:10.1172/JCI164223)
Supplement: Supplemental data [file jci-133-164223-s018.pdf]

Supplemental Methods

Brightfield and fluorescence imaging

Images were taken with Leica TCS SP8 (405/488/552/638 nm) and TCS SPE laser confocal (fluorescence) and DMi8 widefield (equipped with a Leica DFC7000 GT and DMC 4500 camera, brightfield) microscopes with 10x, 20x, 40x or 63x objectives. The diameter range for counting SMI312$^+$ axons in Fiji was set from 0.0 to 8.0 µm^2 (mouse SC; cervical dorsal funiculus) and from 0.0 to 5.0 µm^2 in the (mouse ON). Image analysis of primary somatosensory and barrel field (S1BF) cortical regions (S1BF for AIS length measurement) was carried out on a C2 Nikon confocal microscope (Nikon Instruments, laser lines: 642, 543, and 488 nm). AIS length was measured using a custom morphometrical software AISuite (github.com/jhnnsrs/aisuite2 (1, 2).

High resolution confocal and STED imaging

Tissue sections were transferred in ice-cold methanol or 0.05% triton X-100 for 30 min and rehydrated in PBS for 30 min and blocked afterwards with 1% bovine serum albumin (BSA) for 1h. For 2-color staining (Figure 1A), primary antibodies incubated over night at 4°C and after washing, secondary antibodies (anti-rabbit STAR580, ST580-1002-500UG, Abberior; anti-mouse STAR635P, ST635P-1001-500UG, Abberior) incubated for 2h. For 3-color staining (Figure 1, A and C), samples were incubated with anti-rabbit STAR580 (ST580-1002-500UG, Abberior) and anti-mouse STAR635P (Abberior, Cat. ST635P-1001-500UG) in PBS-T for 2h at RT; samples were incubated with anti-Kir4.1 extracellular antibodies (3) overnight at 4°C, washed, and then incubated with anti-rat AlexaFluor594 (ab150160, Abcam) for 2h. For mouse staining (Figure 1A), primary antibodies pre-coupled to secondary nanobodies (FluoTag-X2 IgG1-STAR635P, N2002-Ab635P-S; FluoTag-X4 IgG-Atto488, N2404-At488-S, NanoTag Biotechnologies) incubated for 2h. For human 3-color staining

(Figure 1C), detection of Kv7.2 was performed with regular primary and secondary antibodies (STAR GREEN, STGREEN-1002-500UG, Abberior) sequentially to Kir4.1 staining. Pre-coupled CASPR antibodies were incubated with STAR GREEN secondaries. All samples were mounted in Dabco-supplemented mowiol and imaged on an Abberior Expert Line (Abberior Instruments) built on a motorized inverted microscope IX83 (Olympus), equipped with pulsed STED lasers (595 nm, 775 nm) shaped by Spatial Light Modulators and excitation lasers (355 nm, 405 nm, 485 nm, 561 nm, and 640 nm). Spectral detection was performed with avalanche photodiodes in following spectral windows: 495-540 nm for STAR GREEN/Atto488, 600-630 nm for STAR580, and 650-725 nm for STAR635P. Images were taken with a 100x/1.40 UPlanSApo Oil immersion objective lens (Olympus) with pixel sizes of 80 nm (confocal) and 30 nm (STED).

Immunoelectron microscopy

Sections were incubated with an anti-Kv7.2 antibody (368103, Synaptic Systems) and detected with protein A-gold (10 nm) obtained from the Cell Microscopy Center, Department of Cell Biology, University Medical Center Utrecht. The sections were analysed using a LEO EM912AB (Zeiss); digital micrographs were taken using a 2048x2048 CCD camera (TRS) (4).

Flow cytometry of mouse splenocytes

Splenocytes were isolated from EAE mice (peak of disease or 35 dpi) and CNS-invading cells were isolated at the same timepoints as described before (5). Mice were perfused transcardially with PBS to diminish contamination by leukocytes in the blood vessels. CNS tissue was dissociated and mononuclear cells from the interface of a 30–50% Percoll (Amersham) density centrifugation were counted by Casy Model TT.

Immune cell subsets of EAE mice were stained in PBS containing 0.5 mM EDTA and 0.1% BSA for 30 min. Fluorescence-labeled monoclonal antibodies (BioLegend): CD11b FITC (N418), CD45R/B220 PE (RA3-6B2), CD11b PerCP/Cy 5.5. (M1/70), NK1.1 APC (PK136), CD8a AF700 (53-6.7), CD45 APC/Cy7 (30-F11), CD4 PB (RM4-5), CD3 BV510 (17A2). Corresponding isotype controls were used for all stainings. $CD4^+$ T cells ($CD45^{high}CD3^+CD4^+CD8^-$), $CD8^+$ T cells ($CD45^{high}CD3^+CD4^-CD8^+$), B cells ($CD45^{high}CD3^-B220^+$), NK cells ($CD45^{high}CD3^-NK1.1^+$), monocytes and macrophages; ($CD45^{high}CD3^-NK1.1^-CD11b^+CD11c^-$), dendritic cells ($CD45^{high}CD3^-NK1.1^-CD11b^+CD11c^+$), microglia ($CD45^{low}CD3^-NK1.1^-CD11b^+CD11c^-$).

Flow cytometry of human and mouse retinal nuclei

Retinal samples (human, mouse) were processed following an analogous procedure. Two animals were pooled for each experiment, whereas each human eye was treated as individual sample. Nuclei were isolated using an RNAse-free lysis buffer containing (in mM): Tris, 10, pH 8; NaCl, 14.6; $MgCl_2$, 8.4; $CaCl_2$, 1; NP40 0.5%. Tissues were homogenized (Wheaton Dounce Tissue Grinder in lysis buffer) and spun down at 500 g for five minutes. Pelleted nuclei were resuspended at a concentration of 8 million nuclei per 100 µl and incubated with a primary labelled antibody against NeuN (ab190565, Abcam, 1:500). Nuclei were visualized by propidium iodide (421301, Biolegend, 1:2,000) and sorted by fluorescence-activated cell sorting on a BD FACSAriaTM III Cell Sorter (BD Biosciences) according to their NeuN fluorescence intensity ($NeuN_{negative}$, $NeuN_{weak}$ and $NeuN_{high}$ nuclei). Sorted groups corresponded to photoreceptors and non-neuronal cells ($NeuN_{negative}$), interneurons ($NeuN_{weak}$) and RGCs ($NeuN_{high}$). RNA was purified with RNeasy Mini Kit (Qiagen) and cDNA synthetized with RevertAid H Minus First Strand cDNA Synthesis Kit (Thermo Fisher Scientific) according to the manufacturer´s instructions.

Quantitative real-time polymerase chain reaction

For quantitative real-time PCR, we used the following TaqMan Gene Expression Assays (Thermo Fisher Scientific): Mm00548884_m1 (Kcnq3), Mm00440080_m1 (Kcnq2), Mm01226041_m1 (Kcnq5). Mm00607939_s1 (b-actin) was used as an endogenous control. All analyses were performed in triplicates. Samples were measured on a QuantStudio Flex Real-Time PCR System. Gene expression of genes of interest was calculated as 2–ΔCT relative to b-actin.

Two-electrode voltage-clamp analysis in *Xenopus laevis* oocytes

Human Kv7.2 and human Kv7.3 cRNAs were prepared from linearized cDNA according to manufacturer instructions (mMESSAGE mMACHINE TSP6 Transcription Kit #AM1340, T7 Transcription Kit #AM1344, Thermo Fischer). Kv7.2/3 channels were expressed in defolliculated stage V/VI *X. laevis* oocytes (EcoCyte Bioscience). Oocytes were injected with 5 ng cRNA encoding human Kv7.2 plus 5 ng cRNA encoding human Kv7.3, and injected oocytes were incubated for 3 days at 18°C in Barths solution, containing (mM): 88 NaCl, 1 KCl, 0.4 $CaCl_2$, 0.33 $Ca(NO_3)_2$, 0.6 $MgSO_4$, 5 TRIS-HCl, 2.4 $NaHCO_3$, supplemented with 80 mg/l theophylline (Sigma Aldrich), 63 mg/l benzylpenicillin (Roth), 40 mg/l streptomycin (Sigma Aldrich), and 100 mg/l gentamycin (Roth). Two-electrode voltage-clamp recordings were performed using a Turbo Tec 10CX amplifier (NPI electronic), NI USB 6221 DA/AD Interface (National Instruments) and custom software GePulse/Ana (http://users.ge.ibf.cnr.it/pusch/programs-mik.htm). Data were sampled at 1.3 kHz. ND96 contained (mM): 96 NaCl, 4 KCl, 1 $CaCl_2$, 1.8 $MgCl_2$, 5 HEPES, pH = 7.4, served as recording solution. Oocytes were recorded at a holding potential of -80 mV using micro-electrodes, the glass capillaries were filled with 3 M KCl, with a resistance

between 0.5 and 1.5 MΩ. The applied pulse protocol consisted of voltage steps from −100 to +40 mV, for 3 s each in 20 mV increments followed by a step back to −30 mV (1s). Oocytes were continuously superfused with ND96, followed by 4-AP administration (0.1, 1 or 5 mM), titrated to a pH of 7.4. Data acquisition was performed with the GePulse software, the I-V relationship of the channels was assessed in Ana Software and statistics were performed via OriginPro 2022b (OriginLab Corporation).

Measurement of serum NfL levels

Whole blood samples were obtained from EAE mice and the control group by cardiac puncture and incubated for 30 min at RT for clotting. The samples were then centrifuged at 2000 × g for 10 min; serum was collected, and frozen at −80°C. Serum NfL levels were measured with a sensitive sandwich ELISA method (NF-lightTM ELISA kit; UmanDiagnostics), according to the manufacturer's instructions. The detection limit of the assay was 33 pg/mL.

Behavioral tests

NOR testing was performed to evaluate memory skills of EAE mice. Animals were habituated to a squared arena (35x40x40 cm) prior to testing to minimize anxiety and exploration. NOR testing consists of a habituation phase (10 min for exploration of two identical objects), followed by three retrieval phases performed at different time intervals after habituation to evaluate short- (30 min and 1 hour) and long-term memory (24 hours) skills (6). For each retrieval session, one old object was substituted with a novel one (chess pieces). NOR index was calculated by time spent exploring novel and old objects: (time novel)/(time novel + time old). NOR index >0.5 indicates that animals were longer exploring the novel object than the old one, suggesting intact memory skills; an index = 0.5 indicates that animals explore both objects for the same

time, suggesting their inability to distinguish between novel and old objects, indicating cognitive impairment (7).

Optical coherence tomography in mice

Retinal *in vivo* imaging using OCT was performed as previously described (8). All experiments were performed under blinding with respect to mouse genotype and treatment conditions.

Visual evoked potentials

Visual pathway conduction was evaluated by recording of flash-light VEPs using an Espion Diagnosys setup (Diagnosys). Mice were anesthetized with xylazine (20 mg/ml, Anased, Akorn) and ketamine (100 mg/ml, Ketathesia, Henry Schein Animal Health) in sterile PBS by i.p. injection and were adapted to darkness for 5 min. Needle electrodes were inserted subcutaneously between the two eyes (active electrode for flash VEP, 8 mm deep), above the tip of the nose (reference) and the tail (ground). 13 min after administration of anesthesia, VEP recordings started and consisted of three runs (3 cd·s/m2, 1 Hz, 4 ms, 6500 K, 100 sweeps). Mouse VEPs resulted in a negative wave after approximately 75 ms (corresponding to human P100) and is referred to as N1 (defined as the first negative deflection after 60 ms). The 2 most reproducible or well-defined waves were used for analysis. All experiments were performed blinded for mouse genotype and treatment condition.

Supplemental figures and legends

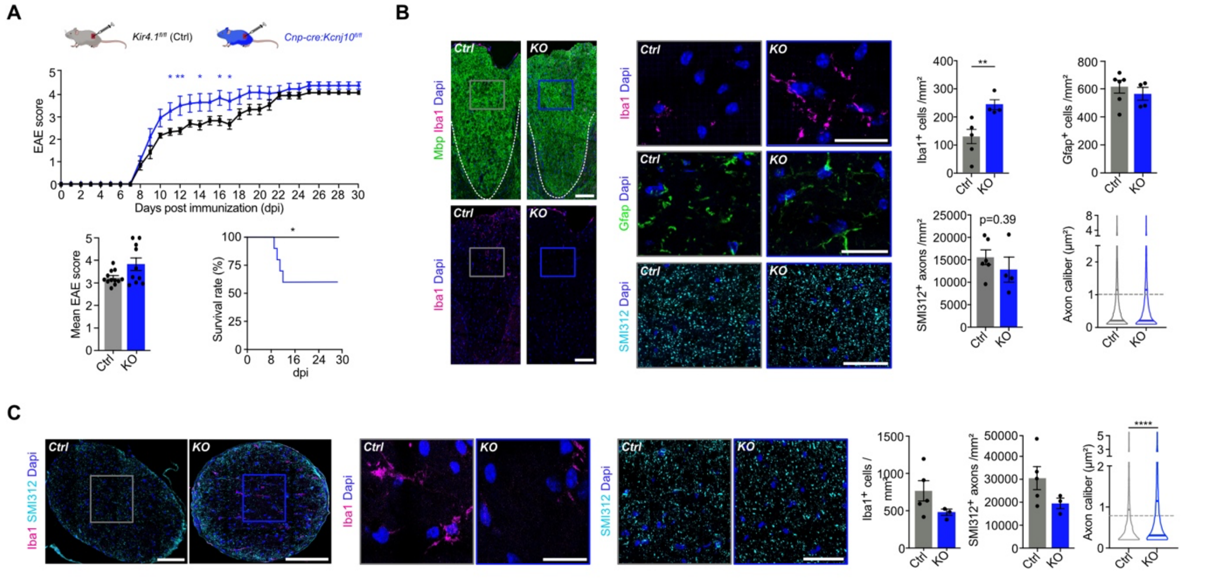

Supplemental Figure 1. Clinical and histological EAE characterization of OL-*Kcnj10* KO mice. (**A**) OL-*Kcnj10* KO mice (n = 10) show more severe EAE symptoms at peak disease than controls (n = 12), but no differences at chronic EAE phase. Loss of OL-*Kcnj10* function increases mortality by 40 % versus EAE in control mice (100% survival). (**B**) OL-*Kcnj10* KO mice (n = 4) exhibit higher density of Iba1$^+$ myeloid cells (IHC) in the SC dorsal funiculus relative to controls (n = 5); Gfap$^+$ astrocyte numbers do not alter between OL-*Kcnj10* KO (n = 4) and control mice (n = 6); SMI312$^+$ axon numbers and calibers do not differ between OL-*Kcnj10* KO (n = 4 mice, n = 14555 axons, mean = 0.9938 µm^2) and controls (n = 6 mice, n = 27347 axons, mean = 1.008 µm^2 represented as dashed gray line). (**C**) No differences comparing OL-*Kcnj10* KO (n = 3) and control mice (n = 5) for Iba1$^+$ myeloid cells as well as for SMI312$^+$ axon numbers based on IHC of ON tissue samples. Note enlarged SMI312$^+$ axons in OL-*Kcnj10* KO mice (n = 3 mice, n = 4917 axons, mean = 0.7461) versus controls (n = 5 mice, n = 6885 axons, mean = 0.6433 µm^2 represented as dashed gray line). Scale bars: (**B**, **C**) 100 µm (overview images), 10 µm (Iba1, Gfap), 50 µm (SMI312). (**A**) Two-way ANOVA with multiple comparisons (up), Mann-Whitney (left), Mantel-Cox (right);

(**B**) Mann-Whitney (Iba1, SMI312 [axon calibers]), unpaired t-test (Gfap, SMI312 [axon density]); (**C**) Unpaired t-tests (Iba1, SMI312 [axon density]), Mann-Whitney (SMI312 [axon calibers]).

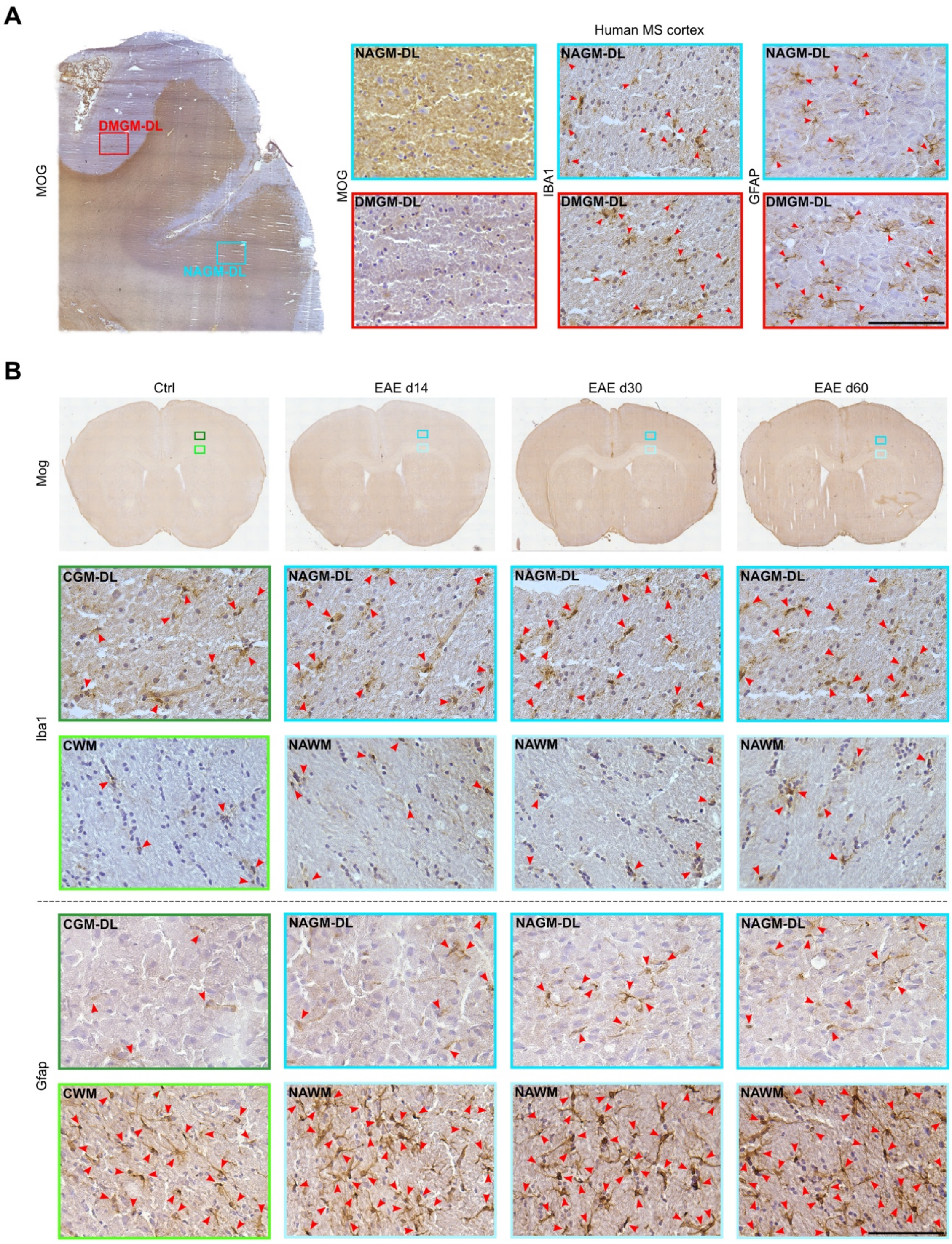

Supplemental Figure 2. Histopathological characterization of human MS and mouse EAE brains. (**A**) MOG IR identifies normal appearing (NAGM) and demyelinated (DMGM) cortical areas from MS brains; representative images of IBA1 and GFAP IR, note all samples analyzed show comparable levels of enhanced microglial reactivity and astrogliosis in DMGM areas (red arrows indicate IBA1^{+}

microglia and GFAP$^+$ astrocytes). (**B**) Mog IR demonstrates absence of cortical DMGM in EAE brains; representative images of Iba1 and Gfap IR showing enhanced glial reactivity and astrogliosis at different EAE disease stages (red arrows indicate Iba1$^+$ microglia and Gfap$^+$ astrocytes). Scale bars: (**A**) 100 μm; (**B**) 20 μM.

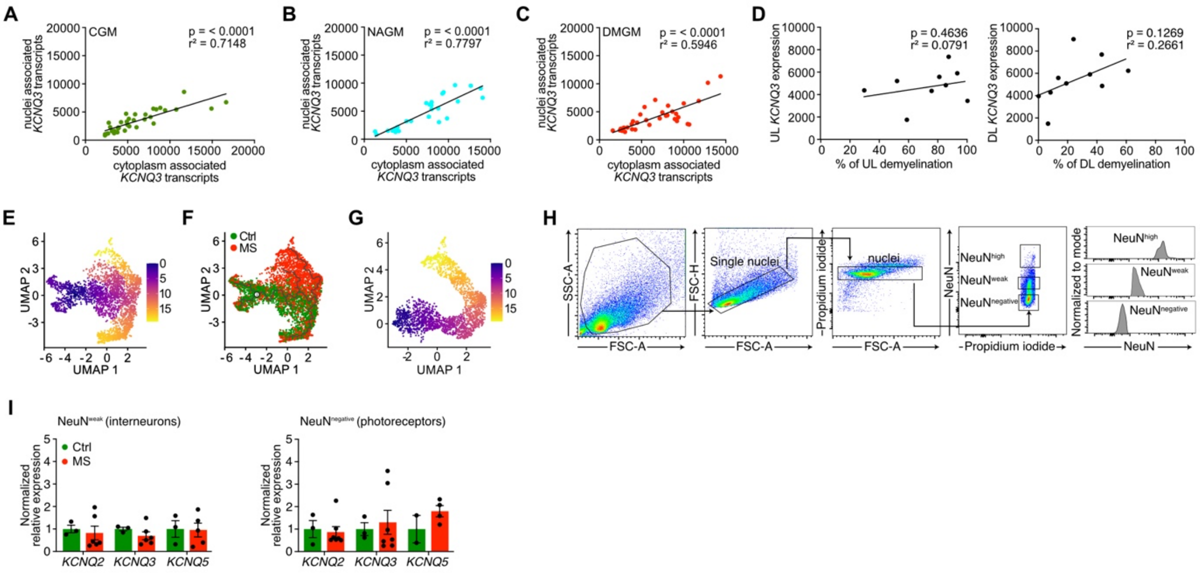

Supplemental Figure 3. Human *KCNQ2/3/5* expression across different tissues. (**A**-**C**) Positive correlation of nucleus- and cytoplasm-associated *KCNQ3* transcript counts (ISH) within the same cell in human cortical tissues in CGM (**A**, n = 35 areas, mean values from 5 patients), NAGM (**B**, n = 27 areas, mean values from 8 samples) and DMGM (**C**, n = 34 areas, mean values from 8 samples). (**D**) Correlation of mean cortical *KCNQ3* expression (ISH) and extent of demyelination (MOG IR) distinguishing between upper and deep cortical layers (each n = 10). (**E**) Unsupervised pseudotime trajectories within L2/3 neurons based on published snRNA-seq dataset (9). (**F**) Distribution of nuclei grouped by condition. (**G**) Unsupervised trajectory inference of upper branch from L2/3 cortical neurons and nuclei distribution along trajectory based on the pseudotime. (**H**) Representative sorting and gating strategy for human retinal nuclei. (**I**) Normalized *KCNQ2*, *KCNQ3* and *KCNQ5* expression (qPCR) measured in sorted human nuclei from neurons comprising interneurons ($NeuN^{weak}$, n = 6) and photoreceptors ($NeuN^{negative}$, controls: n = 6; MS: n = 7). (A-D) Simple linear regression; (I) Mixed-effects model with the Geisser-Greenhouse correction and Sidak's multiple comparison test.

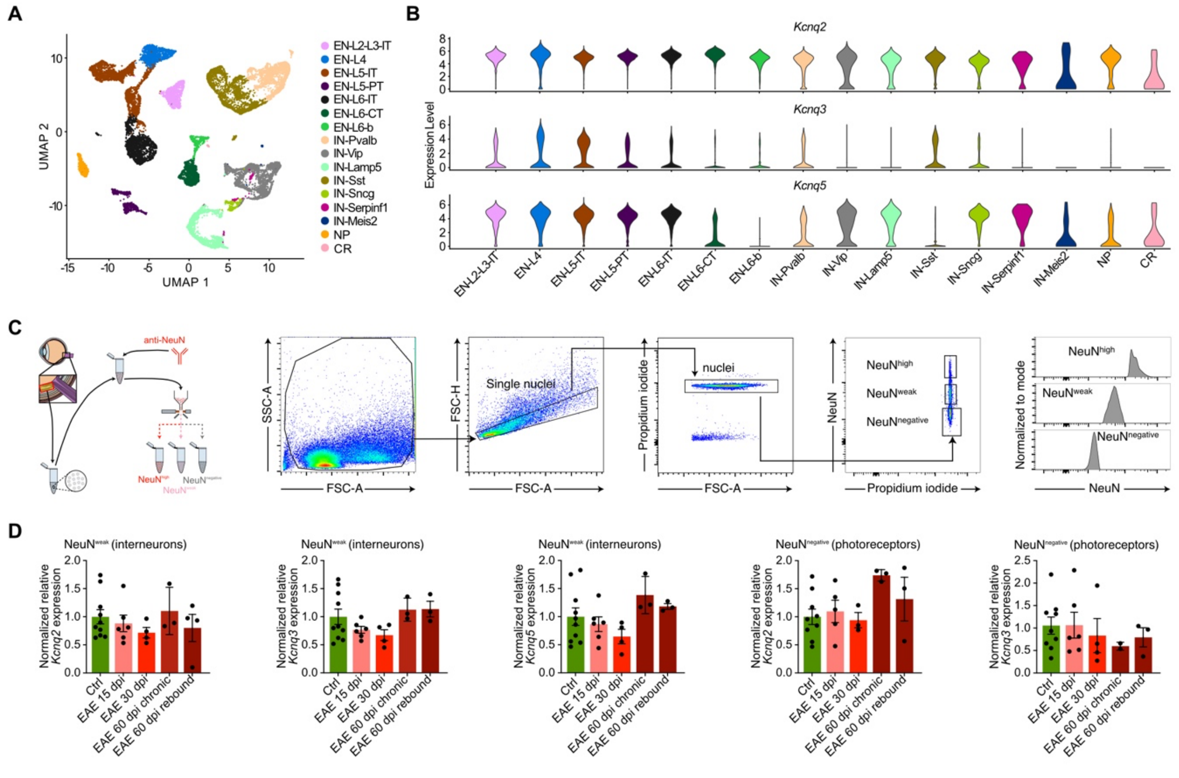

Supplemental Figure 4. Mouse *KCNQ2/3/5* expression across different tissues. (**A**) UMAP plot visualizing 16 cortical mouse cell types derived from published scRNA-seq dataset (10). EN, excitatory neuron; L, layer; IT, intra-telencephalic; PT, pyramidal tract; CT, corticothalamic; IN, inhibitory neuron; NP, near-projecting; CR, Cajal–Retzius cell. (**B**) Normalized *Kcnq2/3/5* expression (scRNA-seq) across different cell types. (**C**) Cartoon illustrates extraction and sorting of retinal nuclei based on NeuN IR. (**D**) Normalized *Kcnq2/3/5* expression (qPCR) in sorted mouse retinal nuclei, NeuN^{weak}: control: n = 10; 15 dpi: n = 6; 30 dpi: n = 4; 60 dpi chronic (no rebound): n = 3; 60 dpi rebound: n = 4 (*Kcnq2*), n = 3 (*Kcnq3/5*) and NeuN^{negative}: control: n = 9; 15 dpi: n = 5 (*Kcnq2*), n = 6 (*Kcnq3*); 30 dpi: n = 3 (*Kcnq2*), n = 4 (*Kcnq3*); 60 dpi chronic: n = 3 (*Kcnq2*), n = 2 (*Kcnq3*); 60 dpi rebound: n = 3). (**D**) One-way ANOVA.

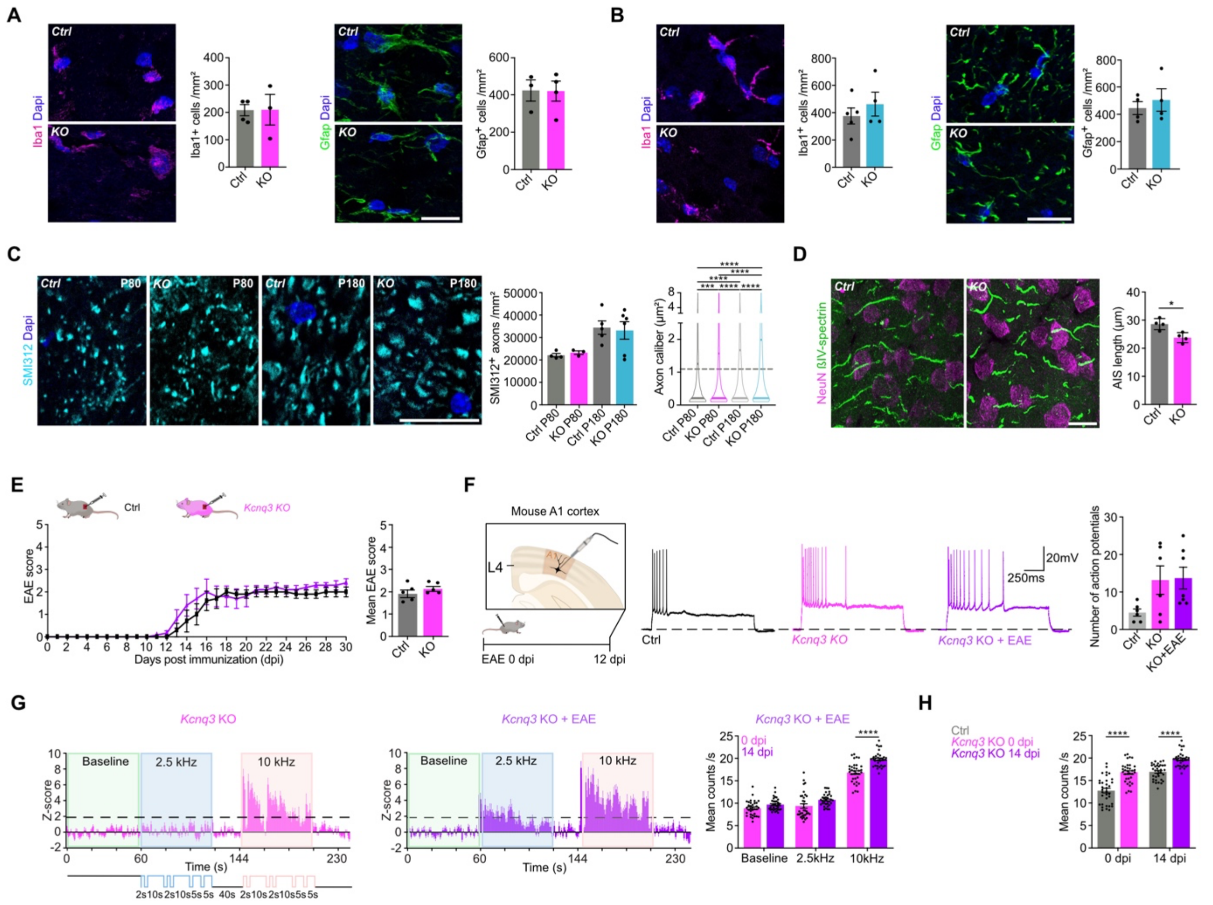

Supplemental Figure 5. Clinical, functional, and histological characterization of *Kcnq3* KO mice. (**A**) P80 *Kcnq3* KO mice (Gfap: n = 4; Iba1: n = 3) show no differences with respect to microglial activation (Iba^{+} myeloid cells) and astrogliosis (Gfap^{+} astrocytes) compared to controls of the same age (Gfap: n = 3; Iba1: n = 4), nor do (**B**) P180 *Kcnq3* KO mice (each n = 4) as compared to their controls (Gfap: n = 4; Iba: n = 5). (**C**) SMI312^{+} axon counts in SC dorsal funiculus in P80 and P180 control (P80: n = 4; P180: n = 5) and *Kcnq3* KO mice (P80: n = 3; P180: n = 6). Note that axon calibers (control P80, n = 18869 axons, mean = 1.095; KO P80, n = 14410 axons, mean = 1.243 represented as scattered gray line; control P180, n = 13535 axons, mean = 1.298; KO P180, n = 13674 axons, mean = 1.437) physiologically enlarge with age and pathologically in KO mice versus controls. (**D**) *Kcnq3* KO mice show decrease in AIS length in layer 2/3 (each n = 4). (**E**) *Kcnq3* KO mice (n = 6) show no altered EAE course versus controls (n = 5). (**F**) *Ex vivo* current-clamp experiments show no altered

excitability in layer 4 neurons of A1 cortex in non-EAE (n = 6) and 12 dpi EAE *Kcnq3* KO mice (n = 78) compared to wildtype controls (n = 6). (**G, H**) *In vivo* recordings show enhanced auditory response to 10 kHz in EAE (14 dpi) *Kcnq3* KO mice versus non-EAE *Kcnq3* KO mice (**G**) and overall, in *Kcnq3* KO mice versus controls (**H**) (each n = 34). Scale bars: (**A**-**D**) 20 µm. (**A**, **B**, **D**) Unpaired t-test; (**C**) Ordinary one-way ANOVA (left), Kruskal-Wallis (right); (**E**) Two-way ANOVA (left), unpaired t-test (right); (**F**) Ordinary one-way ANOVA; (**G**, **H**) Two-way ANOVA.

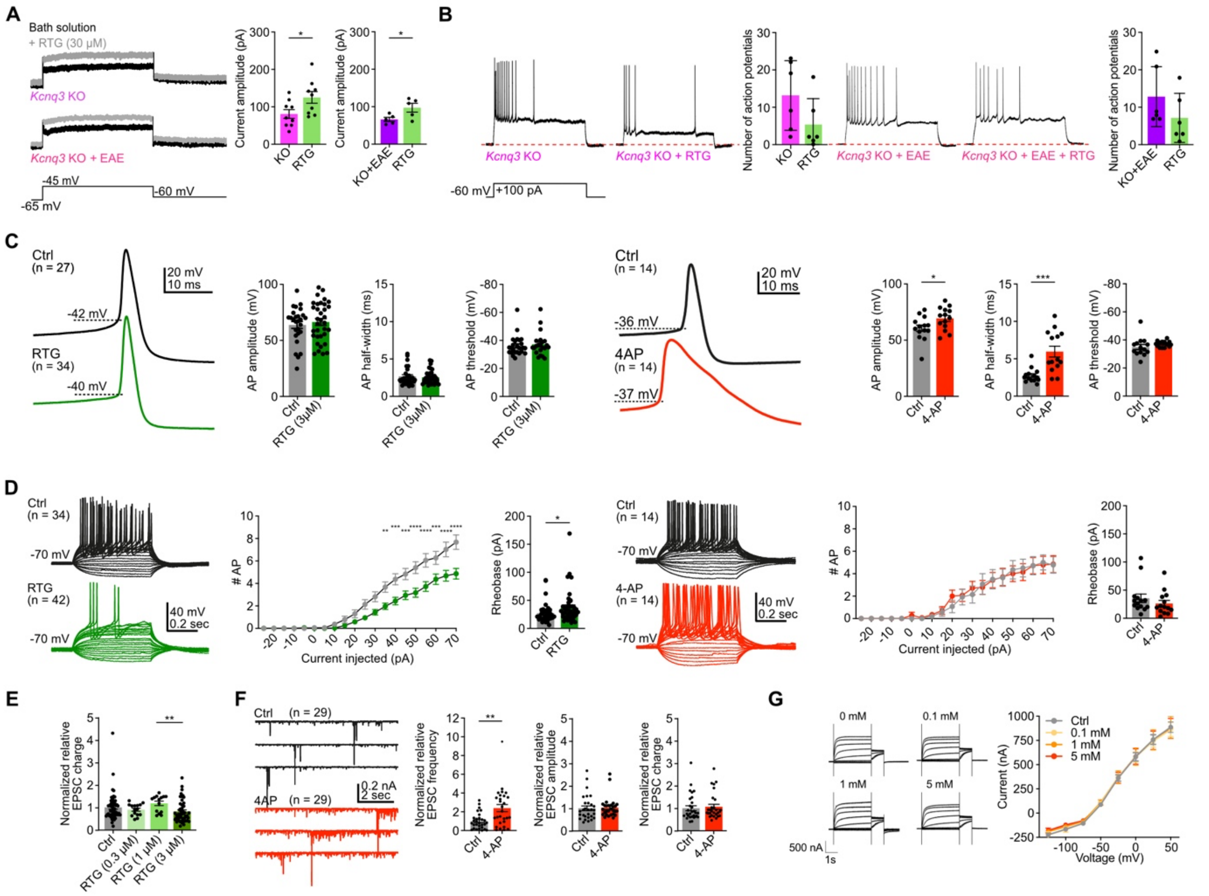

Supplemental Figure 6. Electrophysiological measurements following RTG treatment. (**A**) Voltage-clamp analysis reveals increased M-currents after RTG application (30 µM) in both non-EAE (n = 9) and 12 dpi EAE (n = 5) *Kcnq3* KO brain slices. (**B**) Current-clamp analysis shows an only tending reduction of excitability after RTG application (30 µM) in non-EAE (n = 7), 12 dpi EAE (n = 6) and *Kcnq3* KO brain slices. (**C**) AP traces of RTG- (3 µM) and 4-AP-treated (100 µM) iENs demonstrate that RTG does not affect AP properties (amplitude, half-width: n = 34; threshold: n = 22) compared to control conditions (amplitude, half-width: n = 27; threshold: n = 25), while 4-AP increases AP amplitude and half-width but not AP threshold (each n = 14). (**D**) RTG-treated iENs (3 µM, n = 42) show reduced overall spiking activity in response to current injections and increased rheobase, but no alteration of resting membrane potential compared to untreated iENs (n = 34); 4-AP application (100 µM) had no effect on spiking activity, rheobase and membrane potential compared to control iENs (both

n = 14). (**E**) RTG does not affect normalized relative EPSC charge (0.3 μM, n = 15; 1μM, n = 14; 3 μM, n = 48) compared to control iENs (n = 56). (**F**) Representative EPSC traces show increased normalized relative frequency after 4-AP application (100μM; frequency: n = 29; amplitude, charge: n = 28) compared to control conditions (n = 29). (**G**) Two-electrode voltage clamping reveals no effect of 4-AP treatment (0.1 mM (n = 15); 1 mM (n = 13); 5 mM (n = 18)) on human Kv7.2/Kv7.3 channel function expressed in *X. laevis* oocytes compared to controls (n = 46). (**A**) Unpaired t-test; (**B**, **F**) Mann-Whitney; (**C**) Mann-Whitney (except AP amplitude 4-AP), unpaired t-test (AP amplitude 4-AP); (**D**) Two-way ANOVA (left), Mann-Whitney (right); (**E**) Kruskal-Wallis.

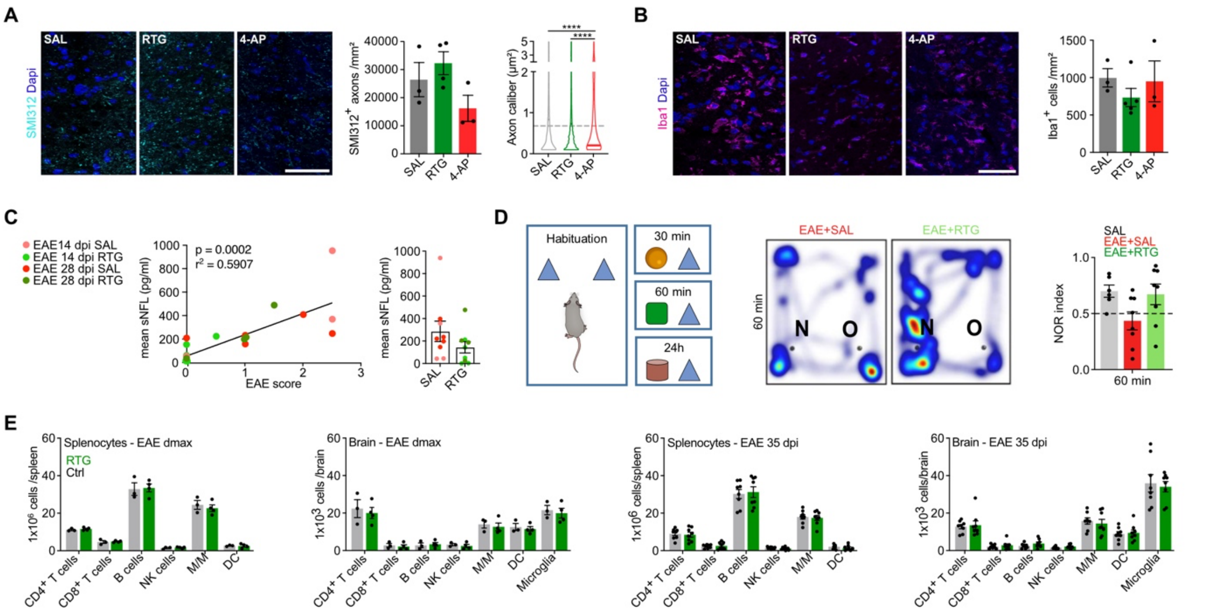

Supplemental Figure 7. RTG treatment effects on peripheral and brain infiltrating immune cells. (**A**) No differences in ON SMI312$^+$ axon counts but axon calibers between SAL- (n = 3, 4432 axons, mean = 0.6115 represented as dashed gray line) and RTG- (n = 4, n = 7238 axons, mean = 0.6322) treated EAE mice compared to 4-AP-treated (n = 3, n = 4004 axons, mean = 0.9594) EAE mice at 30 dpi. (**B**) No difference in number of Iba1$^+$ cells between SAL- (n = 3), RTG- (Iba1: n = 5) and 4-AP-treated (n = 3) EAE mice at 30 dpi. (**C**) Serum NfL levels (EAE at 14 and 28 dpi) correlate with EAE scores and are slightly reduced in RTG-treated (14 dpi: n = 5, 28 dpi: n = 4) versus SAL-treated (14 dpi: n = 4, 28 dpi: n = 5) mice (p = 0.1359). (**D**) Cartoon illustrates NOR test setup to evaluate short- and long-term memory (cf. Figure 4E). RTG (n = 8) versus SAL (n = 9) treatment shows no difference 60 min after habituation and relative to non-EAE (n = 6). (**E**) Absolute EAE immune cell numbers (flow cytometry) at peak disease and at 35 dpi in spleen and brain samples do not differ between RTG versus SAL treatment (1 mg/kg; d_{max}: n = 3 (vehicle), n = 4 (RTG); 35 dpi: n = 8). Scale bars: (**A**, **B**) 50 µm. (**A**) One-way ANOVA (left), Kruskal-Wallis (right); (**B**) Kruskal-Wallis; (**C**) Two-way ANOVA; (**D**) Simple linear regression (left), Mann-Whitney (right).

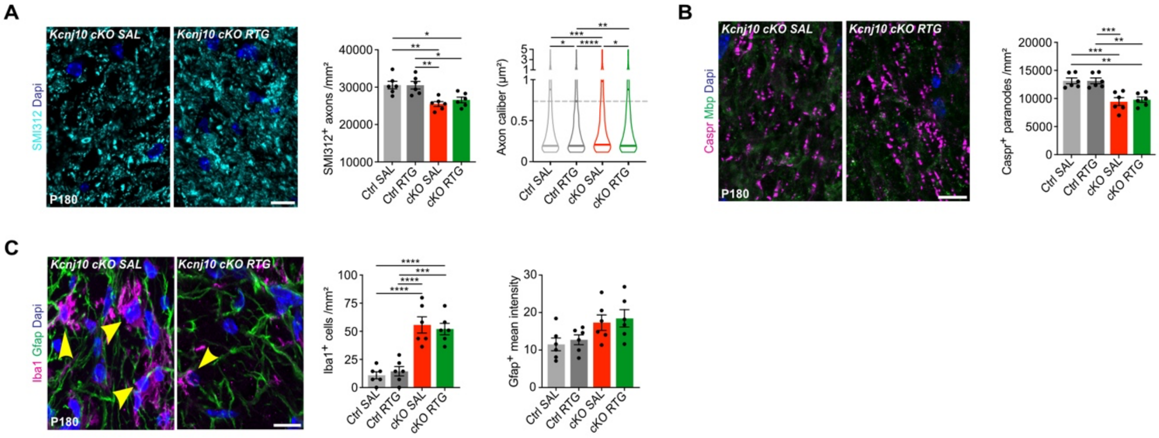

Supplemental Figure 8. Continuous RTG treatment preserves tissue integrity. (**A**) Continuous RTG versus SAL treatment improves SMI312$^+$ axonal pathology in ON samples in OL-*Kcnj10* KO relative to control mice (n = 6 for each group); RTG treatment also prevents axon caliber enlargement in OL-*Kcnj10* KO mice (control SAL: n = 6493 axons, mean = 0.6763 indicated as gray scattered line; control RTG: n = 1869 axons, mean = 0.6024; KO SAL: 4974 axons, mean = 0.7331; KO RTG: 3014 axons, mean = 0.6898; n = 6). (**B**) Continuous RTG versus SAL treatment improves Caspr$^+$ paranode pathology in ON samples in OL-*Kcnj10* KO relative to control mice (n = 6 for each group). (**C**) Continuous RTG versus SAL treatment improves Microglia ON tissue pathology in OL-*Kcnj10* KO relative to control mice (n = 6 for each group). Mean Gfap$^+$ IR was not significantly higher between RTG- and SAL-treated OL-*Kcnj10* KO mice relative to controls (n = 6 for each group). Scale bars: (**A**-**C**) 10µM. (**A**) One-way ANOVA (left), Kruskal-Wallis (right); (**B**-**C**) One-way ANOVA.

Supplemental table legends

Supplemental Table 1

Clinical and pathological data about optic nerve human MS and control tissue samples used for immunohistochemistry.

Supplemental Table 2

Sample-specific clinical metadata for control and MS cortical human samples used for single-nucleus RNA sequencing. The metadata includes information about the percentage of demyelination and subcortical inflammatory lesion stage.

Supplemental Table 3

Clinical and pathological data about cortical human MS and control tissue samples used for RNA *in situ* hybridization and immunohistochemistry.

Supplemental Table 4

Clinical and pathological data about retinal human MS and control tissue samples used for qPCR and RNA *in situ* hybridization.

Supplemental Table 5

Cell-specific metadata (including UMAP coordinates) for human control and MS samples processed using single-nucleus RNA sequencing.

Supplemental Table 6

Results from differential gene expression analysis for *KCNQ2*, *KCNQ3* and *KCNQ5* transcripts in MS and control human cortex samples. The analysis was performed using Wilcoxon rank sum test.

Supplemental Table 7

Differential gene expression analysis of *KCNQ2*, *KCNQ3* and *KCNQ5* of cortical L2/3 neurons (snRNA-seq) in representative MS patients with varying disease duration relative to controls.

Supplemental Table 8

Unsupervised pseudotime trajectory of cortical L2/3 neurons (snRNA-seq) of MS and control cortex tissue samples.

Supplemental Table 9

Differential gene expression analysis of *KCNQ2*, *KCNQ3* and *KCNQ5* in cortical L2/3 neurons (snRNA-seq) grouped by inflammatory lesion stage.

Supplemental Table 10

Cell-specific metadata (including UMAP coordinates) for mouse control cortical samples processed using single-cell RNA sequencing.

References

1. Jamann N, Dannehl D, Lehmann N, Wagener R, Thielemann C, Schultz C, et al. Sensory input drives rapid homeostatic scaling of the axon initial segment in mouse barrel cortex. *Nat Commun.* 2021;12(1):23.
2. Hofflin F, Jack A, Riedel C, Mack-Bucher J, Roos J, Corcelli C, et al. Heterogeneity of the Axon Initial Segment in Interneurons and Pyramidal Cells of Rodent Visual Cortex. *Front Cell Neurosci.* 2017;11:332.
3. Schirmer L, Mobius W, Zhao C, Cruz-Herranz A, Ben Haim L, Cordano C, et al. Oligodendrocyte-encoded Kir4.1 function is required for axonal integrity. *Elife.* 2018;7.
4. Weil MT, Ruhwedel T, Meschkat M, Sadowski B, and Möbius W. Transmission Electron Microscopy of Oligodendrocytes and Myelin. *Methods Mol Biol.* 2019;1936:343-75.
5. Ruck T, Bittner S, Gross CC, Breuer J, Albrecht S, Korr S, et al. CD4+NKG2D+ T cells exhibit enhanced migratory and encephalitogenic properties in neuroinflammation. *PLoS One.* 2013;8(11):e81455.
6. Hammond RS, Tull LE, and Stackman RW. On the delay-dependent involvement of the hippocampus in object recognition memory. *Neurobiol Learn Mem.* 2004;82(1):26-34.
7. Antunes M, and Biala G. The novel object recognition memory: neurobiology, test procedure, and its modifications. *Cogn Process.* 2012;13(2):93-110.
8. Cruz-Herranz A, Balk LJ, Oberwahrenbrock T, Saidha S, Martinez-Lapiscina EH, Lagreze WA, et al. The APOSTEL recommendations for reporting quantitative optical coherence tomography studies. *Neurology.* 2016;86(24):2303-9.

9. Schirmer L, Velmeshev D, Holmqvist S, Kaufmann M, Werneburg S, Jung D, et al. Neuronal vulnerability and multilineage diversity in multiple sclerosis. *Nature.* 2019;573(7772):75-82.

10. Tasic B, Yao Z, Graybuck LT, Smith KA, Nguyen TN, Bertagnolli D, et al. Shared and distinct transcriptomic cell types across neocortical areas. *Nature.* 2018;563(7729):72-8.
